# Supplementary material for: Unpacking equity trends and gaps in Nepal’s progress on maternal health service utilization: Insights from the most recent Demographic and Health Surveys (2011, 2016 and 2022)
Source: PLoS One. 2025 Nov 20;20(11):e0337434. doi: 10.1371/journal.pone.0337434 (PMC12633875; doi:10.1371/journal.pone.0337434)
Supplement: S1 Appendix — (DOCX) [file pone.0337434.s001.docx]

**S1 Appendix.**Supporting information (description of study variables, descriptive analysis of the dependent variables with all outcome variables, descriptive output of trend analysis for all outcome variables, output of bivariable analysis, and crude odds ratios of all outcome variables with independent variables).

Supporting information Table S1: operational definition and categories of the independent variables.

| Characteristics | Categories | Definition |
| --- | --- | --- |
| Maternal Age in Years | 15–19, 20–24, 25–29, 30–34, 35 and above | Age of mothers at the time of interview |
| Religion | Hindus, Other | Other : Buddha, Islam, Kirat, Christian, etc. |
| Ethnicity | Brahmin, Chhettri, Madheshi, Dalit, Janajati, Muslim | Categorized into seven ethnicities, others were merged into the Madheshi caste |
| Education | No Education, Basic, Secondary, Higher | No education, meaning illiterate, classes 1–8 are in basic, and those from class nine and higher are in secondary and higher categories |
| Wealth Quintile | Poorest, Poorer, Middle, Richer, Richest | Five quintiles |
| Disadvantages | Triple (illiterate, poor, and disadvantaged ethnicity), Double (any two of illiterate, poor, and disadvantaged ethnicity), Single (any one of illiterate, poor, and disadvantaged ethnicity), No (rich, literate, and advantaged ethnicity) | This is intersectional variables created using wealth status (rich and poor), ethnicity (disadvantaged and privileged groups), and education (no education and with education) |
| Province | Koshi, Madesh, Bagmati, Gandaki, Lumbini, Karnali, Sudurpaschim | Seven provinces |
| Residence | Urban, Rural | Respondents at the time of the survey live either in rural or urban places |
| Ecological Region | Mountain, Hill, Tarai | Three regions, horizonal division |
| Sex of Child | Male, Female | Sex of the index child |
| Occupation | Not Working, Agriculture, Manual Labor (skilled and unskilled), Working Paid (service and business) | Respondents’ responses are categorized into three groups |
| Language | Nepali, Bhojpuri, Maithili, Other | Individual respondents currently speaking their native languages are categorized into four groups |
| Birth Order | First, Second, Third, Higher | Birth order of a most recent child |

Supporting information Table S2: Trends of Uptake of Routine Maternal Health Services among Women Who Had a Live Birth One Year Prior to the Survey in the Three Most Recent Surveys, by Marginalization Status.

| **4ANC visits** | | | | | | | | | | | | | | | | | |
| --- | --- | --- | --- | --- | --- | --- | --- | --- | --- | --- | --- | --- | --- | --- | --- | --- | --- |
| **Marginalization** | 2011 | | N=1,057 | | 2016 | | N=964 | | 2022 | | N=981 | | p-value  2011-2016 | | p-value 2016-2022 | | p-value 2011-2022 |
| Triple disadvantage | 0.2696 | | 199 | | 0.4998 | | 69 | | 0.6650 | | 82 | | 0.0005 | | 0.0398 | | 0.0000 |
| Double disadvantage | 0.3812 | | 337 | | 0.6135 | | 334 | | 0.7536 | | 293 | | 0.0000 | | 0.0002 | | 0.0000 |
| Single disadvantage | 0.6574 | | 306 | | 0.7483 | | 423 | | 0.8554 | | 426 | | 0.0076 | | 0.0001 | | 0.0000 |
| No disadvantage | 0.8894 | | 215 | | 0.9324 | | 137 | | 0.9131 | | 180 | | 0.1774 | | 0.5269 | | 0.4338 |
| National | 0.5435 | | 1057 | | 0.71 | | 964 | | 0.8198 | | 981 | | 0.0000 | | 0.0000 | | 0.0000 |
| **Institutional delivery** | | | | | | | | | | | | | | | | | |
| **Marginalization** | 2011 | | N=1,057 | | 2016 | | N=964 | | 2022 | | N=981 | | p-value  2011-2016 | | p-value 2016-2022 | | p-value 2011-2022 |
| Triple disadvantage | 0.2256 | | 199 | | 0.4143 | | 69 | | 0.6023 | | 82 | | 0.0025 | | 0.0213 | | 0.0000 |
| Double disadvantage | 0.2895 | | 337 | | 0.4714 | | 334 | | 0.7045 | | 293 | | 0.0000 | | 0.0000 | | 0.0000 |
| Single disadvantage | 0.5707 | | 306 | | 0.7390 | | 423 | | 0.8587 | | 426 | | 0.0000 | | 0.0000 | | 0.0000 |
| No disadvantage | 0.8138 | | 215 | | 0.9677 | | 137 | | 0.9655 | | 180 | | 0.0000 | | 0.9142 | | 0.0000 |
| National | 0.4655 | | 1057 | | 0.6555 | | 964 | | 0.8109 | | 981 | | 0.0000 | | 0.0000 | | 0.0000 |
| **PNC mothers** | | | | | | | | | | | | | | | | | |
| **Marginalization** | 2011 | | N=1,057 | | 2016 | | (N=964) | | 2022 | | N=981 | | p-value  2011-2016 | | p-value 2016-2022 | | p-value 2011-2022 |
| Triple disadvantage | 0.2643 | | 199 | | 0.2877 | | 69 | | 0.5893 | | 82 | | 0.7061 | | 0.00020 | | 0.0000 |
| Double disadvantage | 0.3204 | | 337 | | 0.4456 | | 334 | | 0.6387 | | 293 | | 0.0008 | | 0.0000 | | 0.0000 |
| Single disadvantage | 0.5556 | | 306 | | 0.6260 | | 423 | | 0.7553 | | 426 | | 0.0559 | | 0.0000 | | 0.0000 |
| No disadvantage | 0.8330 | | 215 | | 0.8867 | | 137 | | 0.9036 | | 180 | | 0.1643 | | 0.6249 | | 0.0407 |
| National | 0.4821 | | 1057 | | 0.5763 | | 964 | | 0.7339 | | 981 | | 0.0000 | | 0.0000 | | 0.0000 |
| **COC mothers -all three visits** | | | | | | | | | | | | | | | | | |
| **Marginalization** | 2011 | | N=1,057 | | 2016 | | (N=964) | | 2022 | | N=981 | | p-value  2011-2016 | | p-value 2016-2022 | | p-value 2011-2022 |
| Triple disadvantage | 0.0865 | | 199 | | 0.1841 | | 69 | | 0.3922 | | 82 | | 0.0265 | | 0.0053 | | 0.0000 |
| Double disadvantage | 0.1431 | | 337 | | 0.2712 | | 334 | | 0.4874 | | 293 | | 0.0000 | | 0.0000 | | 0.0000 |
| Single disadvantage | 0.3733 | | 306 | | 0.4919 | | 423 | | 0.6139 | | 426 | | 0.0015 | | 0.0004 | | 0.0000 |
| No disadvantage | 0.7219 | | 215 | | 0.8367 | | 137 | | 0.7969 | | 180 | | 0.0130 | | 0.3669 | | 0.0839 |
| National | 0.3168 | | 1057 | | 0.4424 | | 964 | | 0.5913 | | 981 | | 0.0000 | | 0.0000 | | 0.0000 |
| **CS deliveries** | | | | | | | | | | | | | | | | | |
| **Marginalization** | | 2011 | | N=492 | | 2016 | | N=632 | | 2022 | | N=796 | | p-value  2011-2016 | | p-value 2016-2022 | p-value 2011-2022 |
| Triple disadvantage | | 0.0652 | | 45 | | 0.1334 | | 29 | | 0.1192 | | 49 | | 0.3216 | | 0.8543 | 0.3687 |
| Double disadvantage | | 0.0851 | | 98 | | 0.1005 | | 158 | | 0.1588 | | 206 | | 0.6824 | | 0.105 | 0.0789 |
| Single disadvantage | | 0.1051 | | 175 | | 0.1691 | | 313 | | 0.2564 | | 366 | | 0.0549 | | 0.0059 | 0.0000 |
| No disadvantage | | 0.1951 | | 175 | | 0.2905 | | 133 | | 0.2496 | | 174 | | 0.0509 | | 0.4223 | 0.2208 |
| National | | 0.1295 | | 492 | | 0.1759 | | 632 | | 0.2496 | | 796 | | 0.0332 | | 0.0008 | 0.0000 |
| **Uptake of maternal incentives** | | | | | | | | | | | | | | | | | |
| **Marginalization** | | 2011 | | N=492 | | 2016 | | N=595 | | 2022 | | N=796 | | p-value  2011-2016 | | p-value 2016-2022 | p-value 2011-2022 |
| Triple disadvantage | | 0.7265 | | 45 | | 0.8512 | | 27 | | 0.6805 | | 49 | | 0.2212 | | 0.1039 | 0.6260 |
| Double disadvantage | | 0.7140 | | 98 | | 0.7857 | | 144 | | 0.7381 | | 206 | | 0.2020 | | 0.3064 | 0.6581 |
| Single disadvantage | | 0.8094 | | 175 | | 0.7572 | | 291 | | 0.6386 | | 366 | | 0.1900 | | 0.0011 | 0.0001 |
| No disadvantage | | 0.6529 | | 175 | | 0.6378 | | 132 | | 0.6657 | | 174 | | 0.7841 | | 0.6114 | 0.8008 |
| National | | 0.7273 | | 492 | | 0.742 | | 594 | | 0.6729 | | 796 | | 0.5846 | | 0.0053 | 0.0396 |

Supporting information Table S3: institutional delivery among women aged 15–49 years who had a live birth one year prior to the survey by background variables, NDHS 2022.

| Characteristics | Yes (%) | 95% CIs | N | P-value |
| --- | --- | --- | --- | --- |
| National | 81.1 | 77.8–84 | 981 |  |
| Occupation |  |  |  | 0.03 |
| Not working | 81.7 | 76.8–85.7 | 440 |  |
| Agriculture | 77.6 | 73–81.6 | 398 |  |
| Manual labor | 83.3 | 68.7–91.8 | 57 |  |
| Working paid | 92.9 | 83.6–97.1 | 86 |  |
| Religion |  |  |  | 0.494 |
| Hindu | 81.5 | 78–84.6 | 822 |  |
| Other | 78.8 | 70–85.5 | 160 |  |
| Ethnicity |  |  |  | <0.001 |
| Brahmin | 90.8 | 81.8–95.6 | 81 |  |
| Chhetri | 90.3 | 86.1–93.3 | 183 |  |
| Madhesi | 71.4 | 62.6–78.9 | 167 |  |
| Dalit | 73.3 | 62.5–81.9 | 169 |  |
| Janajati | 85.2 | 79.6–89.5 | 313 |  |
| Muslim | 68.7 | 49.7–83 | 68 |  |
| Education |  |  |  | <0.001 |
| No education | 64.3 | 54.3–73.1 | 161 |  |
| Basic | 73 | 66.8–78.5 | 328 |  |
| Secondary | 91 | 87.6–93.6 | 441 |  |
| Higher | 100 |  | 51 |  |
| Wealth index |  |  |  | <0.001 |
| Poorest | 71.6 | 65.5–77 | 200 |  |
| Poorer | 72.4 | 64.8–78.9 | 212 |  |
| Middle | 82.5 | 75.7–87.7 | 197 |  |
| Richer | 84.9 | 77.4–90.2 | 198 |  |
| Richest | 96.7 | 92–98.7 | 174 |  |
| Disadvantages |  |  |  | <0.001 |
| Triple | 60.2 | 47.2–72 | 82 |  |
| Double | 70.4 | 64.1–76.1 | 293 |  |
| Single | 85.9 | 81.7–89.2 | 426 |  |
| No | 96.6 | 91.6–98.6 | 180 |  |
| Province |  |  |  | <0.001 |
| Koshi | 81.9 | 74.1–87.7 | 188 |  |
| Madhesh | 64.8 | 56.9–71.9 | 239 |  |
| Bagmati | 94.4 | 88.8–97.3 | 160 |  |
| Gandaki | 88.2 | 80–93.3 | 66 |  |
| Lumbini | 84.1 | 72.8–91.3 | 156 |  |
| Karnali | 79.2 | 69.5–86.4 | 70 |  |
| Sudurpashchim | 89.1 | 83.4–92.9 | 102 |  |
| Residence |  |  |  | 0.143 |
| Urban | 82.7 | 78.2–86.4 | 648 |  |
| Rural | 78.1 | 73.2–82.3 | 333 |  |
| Ecological region |  |  |  | 0.143 |
| Mountain | 78.4 | 63.9–88.1 | 60 |  |
| Hill | 85.1 | 80.5–88.7 | 342 |  |
| Terai | 79 | 74–83.3 | 579 |  |
| Maternal age |  |  |  | 0.345 |
| Less than 20 | 79.4 | 72.3–85.1 | 185 |  |
| 20–24 | 81.7 | 76.8–85.8 | 361 |  |
| 25–29 | 78.6 | 72.2–83.9 | 271 |  |
| 30–34 | 87.7 | 79.9–92.7 | 125 |  |
| 35 and above | 79.2 | 62.6–89.6 | 39 |  |
| Native language |  |  |  | <0.001 |
| Nepali | 91.4 | 88.1–93.8 | 476 |  |
| Maithili | 64.1 | 54.6–72.6 | 184 |  |
| Bhojpuri | 65.6 | 54.2–75.4 | 83 |  |
| Other | 79.1 | 72.1–84.8 | 237 |  |
| Birth order |  |  |  | <0.001 |
| First | 91.3 | 87.5–94 | 400 |  |
| Second | 80.5 | 75.4–84.8 | 361 |  |
| Third or higher | 63.5 | 56.1–70.4 | 220 |  |
| At least four ANC visits |  |  |  | <0.001 |
| No | 63.7 | 55.6–71.1 | 177 |  |
| Yes | 84.9 | 81.7–87.7 | 804 |  |

Supporting information Table S4: delivery in private HFs among women aged 15–49 years who had a live birth one year prior to the survey by background variables, NDHS 2022.

| Characteristics | Private HFs (%) | 95% CI | N | P-value |
| --- | --- | --- | --- | --- |
| National | 21.6 | 17.7–26.2 | 796 |  |
| Age in Years |  |  |  | 0.421 |
| <20 | 20.8 | 14.2–29.5 | 147 |  |
| 20–24 | 20.2 | 15.2–26.4 | 295 |  |
| 25–29 | 21 | 15.1–28.4 | 213 |  |
| 30–34 | 29.8 | 18.3–44.6 | 110 |  |
| 35 and above | 15.1 | 5.9–33.4 | 31 |  |
| Religion |  |  |  | 0.555 |
| Hindu | 21.1 | 17.1–25.9 | 670 |  |
| Other | 24.3 | 15.3–36.3 | 126 |  |
| Ethnicity |  |  |  | 0.025 |
| Brahmin | 17.2 | 8.5–31.7 | 74 |  |
| Chhetri | 16.3 | 9.2–27.2 | 165 |  |
| Madhesh | 30.8 | 21.8–41.7 | 119 |  |
| Dalit | 16.3 | 10.1–25.2 | 124 |  |
| Janajati | 21.2 | 14.9–29.1 | 267 |  |
| Muslim | 41.2 | 26–58.2 | 47 |  |
| Education |  |  |  | 0.026 |
| No Education | 13.6 | 8.2–21.8 | 104 |  |
| Basic | 18.6 | 12.9–25.9 | 239 |  |
| Secondary | 22.9 | 18.2–28.4 | 401 |  |
| Higher | 42.5 | 21.3–66.8 | 51 |  |
| Wealth Quintile |  |  |  |  |
| Poorest | 12.5 | 7.8–19.4 | 143 |  |
| Poorer | 10.5 | 6.6–16.5 | 154 |  |
| Middle | 27.6 | 20.3–36.5 | 163 |  |
| Richer | 23.6 | 16.2–33.1 | 168 |  |
| Richest | 31.8 | 21.6–44 | 168 |  |
| Disadvantages |  |  |  | 0.055 |
| Triple | 11.6 | 5.3–23.6 | 49 |  |
| Double | 14.9 | 10.3–21.1 | 206 |  |
| Single | 24.7 | 19.5–30.9 | 366 |  |
| No | 26 | 16.4–38.5 | 174 |  |
| Province |  |  |  | <0.001 |
| Koshi | 35.7 | 25–47.9 | 154 |  |
| Madhesh | 30.7 | 22.1–40.9 | 155 |  |
| Bagmati | 22.6 | 11.9–38.7 | 151 |  |
| Gandaki | 9 | 3.9–19.6 | 58 |  |
| Lumbini | 17.5 | 11.5–25.8 | 131 |  |
| Karnali | 0.6 | 0.1–4.4 | 55 |  |
| Sudurpashchim | 7.7 | 4.2–13.8 | 91 |  |
| Residence |  |  |  | 0.536 |
| Urban | 22.5 | 17.3–28.6 | 536 |  |
| Rural | 19.9 | 14.7–26.4 | 260 |  |
| Ecological Region |  |  |  | 0.002 |
| Mountain | 3.2 | 0.7–13.6 | 47 |  |
| Hill | 15.6 | 9.6–24.2 | 291 |  |
| Terai | 27.4 | 22.2–33.4 | 457 |  |
| Occupation |  |  |  | 0.027 |
| Not Working | 25.1 | 19.4–31.8 | 359 |  |
| Agriculture | 17.3 | 12.9–22.9 | 309 |  |
| Manual Labor | 10.7 | 4.3–24.5 | 48 |  |
| Working Paid | 29.4 | 18.5–43.3 | 80 |  |
| Native Language |  |  |  | 0.002 |
| Nepali | 19 | 14–25.4 | 435 |  |
| Maithili | 38.5 | 28.2–49.9 | 118 |  |
| Bhojpuri | 18.3 | 9.5–32.3 | 55 |  |
| Other | 18 | 12.3–25.6 | 188 |  |
| Birth Order |  |  |  | 0.11 |
| First | 23.9 | 18.9–29.9 | 365 |  |
| Second | 22.8 | 16.4–30.8 | 291 |  |
| Third or Higher | 13.3 | 7.6–22.1 | 140 |  |
| At Least Four ANC Visits |  |  |  | 0.642 |
| No | 23.7 | 15.1–35.3 | 113 |  |
| Yes | 21.3 | 17.2–26.1 | 683 |  |

Supporting information Table S5: Delivery by CS among Women Aged 15–49 Years Who Had a Live Birth at HFs One Year Prior to the Survey by Background Variables, NDHS 2022.

| Characteristics | Delivery by CS (Yes%) | 95%CI | N | P-value |
| --- | --- | --- | --- | --- |
| National | 25 | 21.4–28.9 | 796 |  |
| Age in Years |  |  |  | <0.001 |
| Less than 20 | 10.5 | 6.4–16.9 | 147 |  |
| 20–24 | 21.3 | 16.4–27.2 | 295 |  |
| 25–29 | 32.2 | 24.9–40.6 | 213 |  |
| 30–34 | 35.9 | 25.7–47.6 | 110 |  |
| 35 and above | 39.5 | 23.3–58.3 | 31 |  |
| Religion |  |  |  | 0.6 |
| Hindu | 24.5 | 20.5–29 | 670 |  |
| Other | 27.4 | 18.8–38.1 | 126 |  |
| Ethnicity |  |  | 0.429 |  |
| Brahmin | 32.2 | 21.3–45.5 | 74 |  |
| Chhetri | 24.1 | 15.9–34.7 | 165 |  |
| Madheshi | 24.1 | 16.5–33.9 | 119 |  |
| Dalit | 17.7 | 11.6–26.1 | 124 |  |
| Janajati | 27.3 | 21.7–33.7 | 267 |  |
| Muslim | 24.6 | 13.8–39.9 | 47 |  |
| Education |  |  |  | 0.04 |
| No Education | 14.7 | 8.7–23.7 | 104 |  |
| Basic | 20.3 | 15.2–26.7 | 239 |  |
| Secondary | 28.1 | 23.2–33.7 | 401 |  |
| Higher | 42.6 | 20.1–68.5 | 51 |  |
| Wealth Quintile |  |  |  | <0.001 |
| Poorest | 9.9 | 6.1–15.5 | 143 |  |
| Poorer | 14.6 | 9.8–21.2 | 154 |  |
| Middle | 27 | 19.9–35.5 | 163 |  |
| Richer | 28.7 | 21.2–37.5 | 168 |  |
| Richest | 41.6 | 31.2–52.7 | 168 |  |
| Disadvantages |  |  |  | <0.001 |
| Triple | 11.9 | 5.4–24.3 | 49 |  |
| Double | 15.9 | 11.4–21.7 | 206 |  |
| One | 25.6 | 20.4–31.7 | 366 |  |
| No | 38 | 28.3–48.8 | 174 |  |
| Province |  |  |  | <0.001 |
| Koshi | 35.9 | 29–43.4 | 154 |  |
| Madhesh | 21.8 | 14.5–31.5 | 155 |  |
| Bagmati | 37.9 | 26.6–50.6 | 151 |  |
| Gandaki | 22.4 | 12.7–36.4 | 58 |  |
| Lumbini | 20.1 | 13.8–28.2 | 131 |  |
| Karnali | 7.9 | 3.8–15.6 | 55 |  |
| Sudurpashchim | 9.4 | 5.6–15.3 | 91 |  |
| Residence |  |  |  | <0.001 |
| Urban | 29.2 | 24.4–34.5 | 536 |  |
| Rural | 16.3 | 12.7–20.8 | 260 |  |
| Ecological Region |  |  |  | 0.318 |
| Mountain | 16.7 | 6–38.5 | 47 |  |
| Hill | 22.2 | 16.3–29.4 | 291 |  |
| Terai | 27.6 | 22.9–32.8 | 457 |  |
| Occupation |  |  |  | <0.001 |
| Not Working | 30.3 | 24.8–36.5 | 359 |  |
| Agriculture | 14.9 | 11–19.8 | 309 |  |
| Manual Labor | 25.8 | 12.5–45.9 | 48 |  |
| Working Paid | 39.3 | 26.7–53.6 | 80 |  |
| Native Language |  |  |  | 0.083 |
| Nepali | 28.7 | 23.4–34.8 | 435 |  |
| Maithili | 25.8 | 18.7–34.5 | 118 |  |
| Bhojpuri | 14.1 | 5.3–32.2 | 55 |  |
| Other | 18.9 | 13.2–26.2 | 188 |  |
| Birth Order |  |  |  | <0.001 |
| First | 22.5 | 26.4–40.6 | 365 |  |
| Second | 33.1 | 8.9–22.6 | 291 |  |
| Third or Higher | 14.4 | 8.9–22.6 | 140 |  |
| At Least Four ANC Visits |  |  |  | 0.338 |
| No | 20.5 | 13.1–30.7 | 113 |  |
| Yes | 25.7 | 21.7–30.1 | 683 |  |

Supporting information Table S6: uptake of maternity incentives among women aged 15–49 years who had a live birth at HFs one year prior to the survey by background variables, NDHS 2022.

| Characteristics | Yes (%) | 95% CIs | N | P-value |
| --- | --- | --- | --- | --- |
| National | 67.3 | 62.4–71.9 | 796 |  |
| Respondent’s Age |  |  |  | 0.717 |
| Less than 20 | 65.4 | 57.1–72.9 | 147 |  |
| 20–24 | 66.5 | 59.5–72.8 | 295 |  |
| 25–29 | 71.8 | 63.4–78.9 | 213 |  |
| 30–34 | 63.3 | 46.8–77.2 | 110 |  |
| 35 and above | 67.0 | 47.3–82.1 | 31 |  |
| Religion |  |  |  | 0.419 |
| Hindu | 66.6 | 61.5–71.3 | 670 |  |
| Other | 71.2 | 59.6–80.5 | 126 |  |
| Ethnicity |  |  |  | 0.008 |
| Brahmin | 68.3 | 54.7–79.5 | 74 |  |
| Chhetri | 73.8 | 63.5–82 | 165 |  |
| Madheshi | 49.1 | 39–59.4 | 119 |  |
| Dalit | 67.5 | 56.6–76.7 | 124 |  |
| Janajati | 71.6 | 63.3–78.6 | 267 |  |
| Muslim | 63.8 | 46.9–77.9 | 47 |  |
| Education |  |  |  | 0.122 |
| No Education | 75.4 | 65.4–83.2 | 104 |  |
| Basic | 66.9 | 59.2–73.9 | 239 |  |
| Secondary | 67.8 | 62–73 | 401 |  |
| Higher | 48.9 | 26.3–72 | 51 |  |
| Wealth Quintile |  |  |  | 0.012 |
| Poorest | 79.3 | 72.2–85 | 143 |  |
| Poorer | 70.5 | 61.2–78.4 | 154 |  |
| Middle | 69.9 | 61.4–77.2 | 163 |  |
| Richer | 61.8 | 51.9–70.8 | 168 |  |
| Richest | 57.1 | 44.3–69 | 168 |  |
| Disadvantage |  |  |  | 0.274 |
| Triple | 68.0 | 52.5–80.4 | 49 |  |
| Double | 73.8 | 66.5–80 | 206 |  |
| Single | 63.9 | 57.3–70 | 366 |  |
| No | 66.6 | 54.5–76.8 | 174 |  |
| Province |  |  |  | <0.001 |
| Koshi | 49.7 | 37.7–61.7 | 154 |  |
| Madhesh | 52.4 | 42.3–62.3 | 155 |  |
| Bagmati | 68.7 | 52.5–81.3 | 151 |  |
| Gandaki | 90.4 | 78.2–96.1 | 58 |  |
| Lumbini | 71.7 | 61.4–80.2 | 131 |  |
| Karnali | 85.3 | 78.5–90.3 | 55 |  |
| Sudurpashchim | 88.2 | 81–92.9 | 91 |  |
| Residence |  |  |  | 0.49 |
| Urban | 66.3 | 59.6–72.4 | 536 |  |
| Rural | 69.3 | 63.3–74.8 | 260 |  |
| Ecological Region |  |  |  | <0.001 |
| Mountain | 83.5 | 73.5–90.2 | 47 |  |
| Hill | 76.2 | 67.4–83.2 | 291 |  |
| Terai | 60.0 | 53.5–66.1 | 457 |  |
| Occupation |  |  |  | 0.103 |
| Not Working | 62.7 | 55.8–69.2 | 359 |  |
| Agriculture | 72.7 | 66.7–78 | 309 |  |
| Manual Labor | 73.1 | 54.7–85.9 | 48 |  |
| Working Paid | 63.5 | 49.4–75.6 | 80 |  |
| Native Language |  |  |  | <0.001 |
| Nepali | 70.8 | 63.7–77.1 | 435 |  |
| Maithili | 47.3 | 36.7–58.1 | 118 |  |
| Bhojpuri | 59.1 | 41.9–74.3 | 55 |  |
| Other | 74.1 | 66.3–80.6 | 188 |  |
| Birth Order |  |  |  | 0.258 |
| First | 63.9 | 57.8–69.6 | 365 |  |
| Second | 68.9 | 60.2–76.4 | 291 |  |
| Third or Higher | 72.9 | 63.1–80.8 | 140 |  |
| At Least Four ANC Visits |  |  |  | 0.069 |
| No | 58.8 | 47.5–69.3 | 113 |  |
| Yes | 68.7 | 63.6–73.3 | 683 |  |

Supporting information Table S7: Bivariable analysis of institutional delivery among women who had a live birth in the 1 year prior to the survey by background variables, 2022 Nepal DHS

| Variable | Categories | Crude odds ratio | 95% CI |
| --- | --- | --- | --- |
| Ethnicity | Brahmin | 1.00 |  |
|  | Chhetri | 0.94 | 0.40–2.24 |
|  | Madheshi | 0.25** | 0.10–0.61 |
|  | Dalit | 0.28** | 0.11–0.72 |
|  | Janajati | 0.58 | 0.24–1.41 |
|  | Muslim | 0.22** | 0.07–0.68 |
| Age in years | <20 | 1.00 |  |
|  | 20–24 | 1.16 | 0.72–1.85 |
|  | 25–29 | 0.95 | 0.59–1.54 |
|  | 30–34 | 1.84 | 0.91–3.71 |
|  | ≥35 | 0.99 | 0.40–2.41 |
| Religion | Hindu | 1.00 |  |
|  | Other than Hindu | 0.84 | 0.51–1.38 |
| Education | No education | 1.00 |  |
|  | Basic | 1.51 | 0.92–2.48 |
|  | Secondary | 5.66*** | 3.20–9.99 |
|  | Higher | 0 | 0 |
| Wealth quintile | Lowest | 1.00 |  |
|  | Second | 1.04 | 0.68–1.59 |
|  | Middle | 1.87* | 1.13–3.08 |
|  | Fourth | 2.23** | 1.28–3.88 |
|  | Highest | 11.73*** | 4.38–31.4 |
| Disadvantages | Triple | 1.00 |  |
|  | Double | 1.57 | 0.87–2.84 |
|  | Single | 4.01*** | 2.20–7.32 |
|  | No | 18.50*** | 6.18–55.41 |
| Province | Koshi | 1.00 |  |
|  | Madhesh | 0.41** | 0.23–0.72 |
|  | Bagmati | 3.71** | 1.54–8.96 |
|  | Gandaki | 1.66 | 0.76–3.59 |
|  | Lumbini | 1.17 | 0.52–2.67 |
|  | Karnali | 0.84 | 0.42–1.67 |
|  | Sudurpaschim | 1.80 | 0.93–3.49 |
| Residence | Urban | 1.00 |  |
|  | Rural | 0.75 | 0.51–1.10 |
| Ecoregion | Mountain | 1.00 |  |
|  | Hill | 1.58 | 0.71–3.47 |
|  | Terai | 1.04 | 0.48–2.25 |
| Occupation | Not working | 1.00 |  |
|  | Agriculture | 0.78 | 0.55–1.11 |
|  | Manual labor | 1.12 | 0.47–2.67 |
|  | Working paid | 2.94* | 1.09–7.93 |
| Native language | Nepali | 1.00 |  |
|  | Maithili | 0.17*** | 0.1–0.29 |
|  | Bhojpuri | 0.18*** | 0.1–0.33 |
|  | Other | 0.36*** | 0.21–0.6 |
| Birth order | First | 1.00 |  |
|  | Second | 0.40*** | 0.24–0.66 |
|  | Third or higher | 0.17*** | 0.10–0.27 |
| At least four antenatal care visits | No | 1.00 |  |
|  | Yes | 3.20*** | 2.17–4.72 |
| * p<.05, ** p<.01, *** p<.001 | | | |

Supporting information Table S8: Bivariable analysis of institutional delivery in private health facilities among women who had a live birth in the 1 year prior to the survey by background variables, 2022 Nepal DHS

| Variable | Categories | Crude odds ratio | 95% CI |
| --- | --- | --- | --- |
| Age in years | <20 | 1.00 |  |
|  | 20–24 | 0.96 | 0.56–1.65 |
|  | 25–29 | 1.01 | 0.56–1.83 |
|  | 30–34 | 1.61 | 0.74–3.49 |
|  | ≥35 | 0.67 | 0.22–2.10 |
| Religion | Hindu | 1.00 |  |
|  | Other than Hindu | 1.20 | 0.66–2.19 |
| Ethnicity | Brahmin | 1.00 |  |
|  | Chhetri | 0.94 | 0.41–2.12 |
|  | Madheshi | 2.15 | 0.83–5.53 |
|  | Dalit | 0.94 | 0.35–2.48 |
|  | Janajati | 1.29 | 0.53–3.16 |
|  | Muslim | 3.37* | 1.16–9.82 |
| Maternal education | No education | 1.00 |  |
|  | Basic | 1.44 | 0.8–2.61 |
|  | Secondary | 1.88* | 1.00–3.52 |
|  | Higher | 4.69** | 1.50–14.66 |
| Wealth quintile | Lowest | 1.00 |  |
|  | Second | 0.82 | 0.42–1.63 |
|  | Middle | 2.67** | 1.47–4.84 |
|  | Fourth | 2.16* | 1.06–4.39 |
|  | Highest | 3.26** | 1.56–6.77 |
| Disadvantage | Triple | 1.00 |  |
|  | Double | 1.34 | 0.53–3.36 |
|  | Single | 2.51* | 1.05–5.99 |
|  | No | 2.67 | 0.95–7.49 |
| Province | Koshi | 1.00 |  |
|  | Madhesh | 0.8 | 0.41–1.57 |
|  | Bagmati | 0.53 | 0.21–1.33 |
|  | Gandaki | 0.18** | 0.06–0.5 |
|  | Lumbini | 0.38** | 0.19–0.78 |
|  | Karnali | 0.01*** | 0.00–0.09 |
|  | Sudurpaschim | 0.15*** | 0.07–0.34 |
| Residence | Urban | 1.00 |  |
|  | Rural | 0.86 | 0.53–1.4 |
| Ecoregion | Mountain | 1.00 |  |
|  | Hill | 5.51* | 1.07 - 28.31 |
|  | Terai | 11.29** | 2.32–54.83 |
| Occupation | Not working | 1 |  |
|  | Agriculture | 0.62* | 0.41–0.96 |
|  | Manual labor | 0.36* | 0.13–0.97 |
|  | Working paid | 1.24 | 0.65–2.36 |
| Native language | Nepali | 1.00 |  |
|  | Maithili | 2.66** | 1.47–4.82 |
|  | Bhojpuri | 0.95 | 0.41–2.21 |
|  | Other | 0.94 | 0.54–1.61 |
| Birth order | First | 1.00 |  |
|  | Second | 0.94 | 0.58–1.50 |
|  | Third or higher | 0.49* | 0.25–0.94 |
| At least four ANC visits | No | 1.00 |  |
|  | Yes | 0.87 | 0.48–1.57 |
| * p<.05, ** p<.01, *** p<.001 | | | |

Supporting information Table S9: Bivariable logistic regression analysis of delivery by cesarean section among women who had a live birth in the 1 year prior to the survey by background variables, 2022 Nepal DHS

| Variable | Categories | Crude odds ratio | 95% CI |
| --- | --- | --- | --- |
| Ethnicity (ref: Brahmin) | Chhetri | 0.67 | 0.34–1.3 |
|  | Madheshi | 0.67 | 0.31–1.42 |
|  | Dalit | 0.45* | 0.21–0.96 |
|  | Janajati | 0.79 | 0.4–1.56 |
|  | Muslim | 0.69 | 0.28–1.7 |
| Education (ref: No education) | Basic | 1.48 | 0.74–2.97 |
|  | Secondary | 2.27* | 1.20–4.32 |
|  | Higher | 4.31* | 1.27–14.58 |
| Wealth quintile (ref: Lowest) | Second | 1.56 | 0.77–3.17 |
|  | Middle | 3.37*** | 1.76–6.45 |
|  | Fourth | 3.67*** | 1.94–6.94 |
|  | Highest | 6.48*** | 3.26–12.91 |
| Disadvantages (ref: Triple) | Double | 1.39 | 0.54–3.63 |
|  | Single | 2.55* | 1.02–6.34 |
|  | No | 4.53** | 1.73–11.83 |
| Province (ref: Koshi) | Madhesh | 0.5* | 0.28–0.90 |
|  | Bagmati | 1.09 | 0.59–1.99 |
|  | Gandaki | 0.51 | 0.24–1.10 |
|  | Lumbini | 0.45** | 0.26–0.78 |
|  | Karnali | 0.15*** | 0.07–0.35 |
|  | Sudurpaschim | 0.18*** | 0.10–0.35 |
| Residence (ref: Urban) | Rural | 0.47*** | 0.32–0.70 |
| Ecoregion (ref: Mountain) | Hill | 1.43 | 0.42–4.85 |
|  | Terai | 1.91 | 0.59–6.15 |
| Occupation (ref: Not working) | Agriculture | 0.40*** | 0.26–0.63 |
|  | Manual labor | 0.80 | 0.32–2.01 |
|  | Working paid | 1.49 | 0.78–2.84 |
| Native language (ref: Nepali) | Maithili | 0.86 | 0.52–1.42 |
|  | Bhojpuri | 0.41 | 0.14–1.22 |
|  | Other | 0.58* | 0.34–0.99 |
| Birth order (ref: First) | Second | 1.71** | 1.14–2.57 |
|  | Third or higher | 0.58 | 0.32–1.05 |
| At least four antenatal care visits (ref: No) | Yes | 1.34 | 0.73–2.45 |
| * p<.05, ** p<.01, *** p<.001 | | | |

Supporting information Table S10: Bivariable logistic regression analysis of uptake of maternity incentives among women who had a live birth in the 1 year prior to the survey by background variables, 2022 Nepal DHS

| Variable | Categories | Crude odds ratio | 95% CI |
| --- | --- | --- | --- |
| Ethnicity (ref: Brahmin) | Chhetri | 1.30 | 0.69–2.47 |
|  | Madheshi | 0.45* | 0.22–0.91 |
|  | Dalit | 0.96 | 0.46–2.01 |
|  | Janajati | 1.17 | 0.60–2.27 |
|  | Muslim | 0.82 | 0.33–2.02 |
| Education (ref: No education) | Basic | 0.66 | 0.38–1.16 |
|  | Secondary | 0.69 | 0.41–1.16 |
|  | Higher | 0.31* | 0.10–0.94 |
| Wealth Quintile (ref: Lowest) | Second | 0.62 | 0.37–1.07 |
|  | Middle | 0.60 | 0.35–1.04 |
|  | Fourth | 0.42** | 0.24–0.74 |
|  | Highest | 0.35** | 0.18–0.66 |
| Disadvantages (ref: Triple) | Double | 1.32 | 0.66–2.65 |
|  | Single | 0.83 | 0.42–1.66 |
|  | No | 0.93 | 0.41–2.13 |
| Province (ref: Koshi) | Madhesh | 1.11 | 0.59–2.11 |
|  | Bagmati | 2.22 | 0.96–5.17 |
|  | Gandaki | 9.49*** | 3.24–27.85 |
|  | Lumbini | 2.57** | 1.31–5.06 |
|  | Karnali | 5.89*** | 3.00–11.56 |
|  | Sudurpaschim | 7.54*** | 3.59–15.84 |
| Residence (ref: Urban) | Rural | 1.15 | 0.77–1.71 |
| Ecoregion (ref: Mountain) | Hill | 0.63 | 0.30–1.34 |
|  | Terai | 0.30*** | 0.15–0.57 |
| Occupation (ref: Not working) | Agriculture | 1.58* | 1.08–2.31 |
|  | Manual labor | 1.61 | 0.73–3.57 |
|  | Working paid | 1.03 | 0.57–1.87 |
| Native language (ref: Nepali) | Maithili | 0.37*** | 0.22–0.63 |
|  | Bhojpuri | 0.59 | 0.28–1.28 |
|  | Others | 1.18 | 0.73–1.89 |
| Birth order (ref: First) | Second | 1.25 | 0.81–1.93 |
|  | Third or higher | 1.52 | 0.92–2.50 |
| At least four antenatal care visits (ref: No) | Yes | 1.54 | 0.96–2.44 |
| * p<.05, ** p<.01, *** p<.001. | | | |
