# Supplementary material for: Unpacking equity trends and gaps in Nepal’s progress on maternal health service utilization: Insights from the most recent Demographic and Health Surveys (2011, 2016 and 2022)
Source: PLoS One. 2025 Nov 20;20(11):e0337434. doi: 10.1371/journal.pone.0337434 (PMC12633875; doi:10.1371/journal.pone.0337434)
Supplement: Attachment — Submitted filename: PONE-D-25-36549-1.pdf [file pone.0337434.s002.pdf]

PLOS ONE

Trends and Determinants of Maternal Health Service Utilization in Nepal: Insights from the Most Recent Three Demographic and Health Surveys

--Manuscript Draft--

**Manuscript Number:**	PONE-D-25-36549
**Article Type:**	Research Article
**Full Title:**	Trends and Determinants of Maternal Health Service Utilization in Nepal: Insights from the Most Recent Three Demographic and Health Surveys
**Short Title:**	Trends and Determinants of Maternal Health Service Utilization in Nepal
**Corresponding Author:**	Resham B Khatri, PhD<br>Health Social Science and Development Research Institute<br>Kathmandu, Bagmati NEPAL
**Keywords:**	Institutional delivery, cesarean section, maternal incentive, private health facilities, health equity, multiple marginalization
**Abstract:**	Background<br>Over the past three decades, Nepal has made significant progress in enhancing access to maternal health services and in reducing maternal morbidity and mortality rates. Nonetheless, the maternal mortality ratio (MMR) remains high, with a slower rate of decline in recent years, particularly among disadvantaged groups. This study investigates trends and determinants of key maternal health services, considering selected socioeconomic and demographic factors within the Nepali context.<br>Methods<br>This study employed trend analyses utilizing data from the Nepal Demographic and Health Surveys (NDHS) conducted in 2011 (n=1,057), 2016 (n=964), and 2022 (n=981) among women aged 15–49 who had experienced at least one live birth prior to each survey. The outcome variables for the trend analysis included the attainment of at least four antenatal care (ANC) visits, institutional deliveries, first postnatal care (PNC) within 48 hours of childbirth, and the completion of all maternal care visits. Determinants of institutional delivery were assessed through multivariable analysis of the NDHS 2022 dataset, focusing on women's characteristics and their disadvantaged status, encompassing intersections of wealth, ethnicity, and education levels.<br>Results<br>Trend analysis across the three NDHS datasets revealed low completion rates and significant discontinuity of care throughout the maternity continuum. There was an observed increase in institutional deliveries and cesarean sections (CS) within private health facilities, alongside a decreasing trend in the utilization of maternity incentives. The multivariable analysis of the NDHS 2022 data indicated that women from Karnali province and those facing multiple forms of disadvantage—including low wealth, disadvantaged ethnicities, lack of education, and higher birth orders—exhibited lower rates of institutional delivery. Conversely, women who attended at least four ANC visits demonstrated higher completion rates. Increased odds of childbirth in private health facilities were identified in the Koshi, Bagmati, Madhesh, and Lumbini provinces, particularly among women with fewer disadvantages. In contrast, women engaged in manual labor or those with higher birth orders had reduced rates of childbirth in private facilities. Notably, higher odds of cesarean sections were observed among older women, Maithili native speakers, and in provinces characterized by elevated rates of private facility deliveries. Furthermore, the uptake of maternity incentives was lower in private health facilities and the Koshi, Bagmati, Madhesh, and Lumbini provinces.<br>Conclusions<br>The findings indicate that women from the most disadvantaged groups experience lower rates of routine maternal care visits and greater discontinuity of care from ANC to PNC, resulting in significant equity gaps compared to their advantaged counterparts. The rising trend of deliveries in private health facilities and cesarean sections, coupled with limited or non-existent maternal incentives, imposes a financial burden on already marginalized populations. Health systems must prioritize designing and implementing targeted strategies tailored to local needs, acknowledging the significance of intersecting disadvantages and their influence on the equity and universality of maternal services in Nepal and other similar settings.

Powered by Editorial Manager® and ProduXion Manager® from Aries Systems Corporation

Order of Authors:	Resham B Khatri, PhD
	Rolina Dhital
	Sabita Tuladhar
	Nisha Joshi Bhatta
	Yibeltal Assefa

Additional Information:	
**Question**	**Response**
**Financial Disclosure**<br><br>Enter a financial disclosure statement that describes the sources of funding for the work included in this submission. Review the submission guidelines for detailed requirements. View published research articles from *PLOS ONE* for specific examples.<br><br>This statement is required for submission and **will appear in the published article** if the submission is accepted. Please make sure it is accurate.<br><br>**Funded studies**<br>Enter a statement with the following details:<br>• Initials of the authors who received each award<br>• Grant numbers awarded to each author<br>• The full name of each funder<br>• URL of each funder website<br>• Did the sponsors or funders play any role in the study design, data collection and analysis, decision to publish, or preparation of the manuscript?<br><br>Did you receive funding for this work?	The author(s) received no specific funding for this work.
**Competing Interests**<br><br>Use the instructions below to enter a competing interest statement for this submission. On behalf of all authors, disclose any competing interests that could be perceived to bias this work—acknowledging all financial support and any other relevant financial or non-financial competing interests.	The authors have declared that no competing interests exist.

Powered by Editorial Manager® and ProduXion Manager® from Aries Systems Corporation

This statement is required for submission and **will appear in the published article** if the submission is accepted. Please make sure it is accurate and that any funding sources listed in your Funding Information later in the submission form are also declared in your Financial Disclosure statement.<br><br>View published research articles from *PLOS ONE* for specific examples.<br><br>**NO authors have competing interests**<br><br>Enter: *The authors have declared that no competing interests exist.*<br><br>**Authors with competing interests**<br><br>Enter competing interest details beginning with this statement:<br><br>*I have read the journal's policy and the authors of this manuscript have the following competing interests: [insert competing interests here]*<br><br>* typeset	
**Ethics Statement**<br><br>Enter an ethics statement for this submission. This statement is required if the study involved:<br><br>• Human participants<br>• Human specimens or tissue<br>• Vertebrate animals or cephalopods<br>• Vertebrate embryos or tissues<br>• Field research<br><br>Write "N/A" if the submission does not require an ethics statement.<br><br>General guidance is provided below. Consult the submission guidelines for detailed instructions. **Make sure that all information entered here is included in the**	N/A.Not applicable. We used publicly accessible, de-identified datasets from the Demographic and Health Survey program (https://dhsprogram.com/data/available-datasets.cfm). The survey received ethical approval from the ICF Institutional Review Board in the USA and the Nepal Health Research Council, Nepal. Mothers provided informed consent both for themselves and their children. Given that this research involved only a secondary analysis of completely anonymised data, there was no need for additional ethical clearance. The author (RBK) received approval to access and utilize the de-identified data.

**Methods section of the manuscript.**<br><br>**Format for specific study types**<br><br>**Human Subject Research (involving human participants and/or tissue)**<br>• Give the name of the institutional review board or ethics committee that approved the study<br>• Include the approval number and/or a statement indicating approval of this research<br>• Indicate the form of consent obtained (written/oral) or the reason that consent was not obtained (e.g. the data were analyzed anonymously)<br><br>**Animal Research (involving vertebrate animals, embryos or tissues)**<br>• Provide the name of the Institutional Animal Care and Use Committee (IACUC) or other relevant ethics board that reviewed the study protocol, and indicate whether they approved this research or granted a formal waiver of ethical approval<br>• Include an approval number if one was obtained<br>• If the study involved *non-human primates*, add *additional details* about animal welfare and steps taken to ameliorate suffering<br>• If anesthesia, euthanasia, or any kind of animal sacrifice is part of the study, include briefly which substances and/or methods were applied<br><br>**Field Research**<br><br>Include the following details if this study involves the collection of plant, animal, or other materials from a natural setting:<br>• Field permit number<br>• Name of the institution or relevant body that granted permission	
**Data Availability**<br><br>Authors are required to make all data underlying the findings described fully available, without restriction, and from the time of publication. PLOS allows rare exceptions to address legal and ethical concerns. See the PLOS Data Policy and FAQ for detailed information.	Yes - all data are fully available without restriction

Powered by Editorial Manager® and ProduXion Manager® from Aries Systems Corporation

A Data Availability Statement describing where the data can be found is required at submission. Your answers to this question constitute the Data Availability Statement and **will be published in the article**, if accepted.<br><br>**Important:** Stating 'data available on request from the author' is not sufficient. If your data are only available upon request, select 'No' for the first question and explain your exceptional situation in the text box.<br><br>Do the authors confirm that all data underlying the findings described in their manuscript are fully available without restriction?	
**Describe where the data may be found in full sentences. If you are copying our sample text, replace any instances of XXX with the appropriate details.**<br><br>• If the data are **held or will be held in a public repository**, include URLs, accession numbers or DOIs. If this information will only be available after acceptance, indicate this by ticking the box below. For example: *All XXX files are available from the XXX database (accession number(s) XXX, XXX.*).<br>• If the data are all contained **within the manuscript and/or Supporting Information files**, enter the following: *All relevant data are within the manuscript and its Supporting Information files.*<br>• If neither of these applies but you are able to provide **details of access elsewhere**, with or without limitations, please do so. For example:<br><br>*Data cannot be shared publicly because of [XXX]. Data are available from the XXX Institutional Data Access / Ethics Committee (contact via XXX) for researchers who meet the criteria for access to confidential data.*<br><br>*The data underlying the results*	All relevant data are within the manuscript and its Supporting Information files.

presented in the study are available from (include the name of the third party and contact information or URL).<br>• This text is appropriate if the data are owned by a third party and authors do not have permission to share the data.<br><br>* typeset	
Additional data availability information:	

Powered by Editorial Manager® and ProduXion Manager® from Aries Systems Corporation

Manuscript

Click here to access/download;Manuscript;0 Manuscript-MH_08.07.2025.docx

Trends and Determinants of Maternal Health Service Utilization in Nepal: Insights from the Most Recent Three Demographic and Health Surveys

Resham B Khatri*[1,2], Rolina Dhital[3,] Sabita Tuladhar[4] Nisha Joshi Bhatta[5], Yibeltal Assefa[2]

[1] Health Social Science and Development Research Institute, Kathmandu, Nepal

[2] School of Public Health, University of Queensland, Brisbane, Australia

[3] Health Action and Research, Kathmandu, Nepal

[4] Nepal Public Health Association, Kathmandu, Nepal.

[5] Family Welfare Division, Ministry of Health and Population, Kathmandu, Nepal

*Corresponding Author:

email: rkchettri@gmail.com.

Abstract

Background

Over the past three decades, Nepal has made significant progress in enhancing access to maternal health services and in reducing maternal morbidity and mortality rates. Nonetheless, the maternal mortality ratio (MMR) remains high, with a slower rate of decline in recent years, particularly among disadvantaged groups. This study investigates trends and determinants of key maternal health services, considering selected socioeconomic and demographic factors within the Nepali context.

Methods

This study employed trend analyses utilizing data from the Nepal Demographic and Health Surveys (NDHS) conducted in 2011 (n=1,057), 2016 (n=964), and 2022 (n=981) among women aged 15–49 who had experienced at least one live birth prior to each survey. The outcome variables for the trend analysis included the attainment of at least four antenatal care (ANC) visits, institutional deliveries, first postnatal care (PNC) within 48 hours of childbirth, and the completion of all maternal care visits. Determinants of institutional delivery were assessed through multivariable analysis of the NDHS 2022 dataset, focusing on women's characteristics and their disadvantaged status, encompassing intersections of wealth, ethnicity, and education levels.

Results

Trend analysis across the three NDHS datasets revealed low completion rates and significant discontinuity of care throughout the maternity continuum. There was an observed increase in institutional deliveries and cesarean sections (CS) within private health facilities, alongside a decreasing trend in the utilization of maternity incentives. The multivariable analysis of the NDHS 2022 data indicated that women from Karnali province and those facing multiple forms of disadvantage—including low wealth, disadvantaged ethnicities, lack of education, and higher birth orders—exhibited lower rates of institutional delivery. Conversely, women who attended at least four ANC visits demonstrated higher completion rates. Increased odds of childbirth in private health facilities were identified in the Koshi, Bagmati, Madhesh, and Lumbini provinces, particularly among women with fewer disadvantages. In contrast, women engaged in manual labor or those with higher birth orders had reduced rates of childbirth in private facilities. Notably, higher odds of cesarean sections were observed among older women, Maithili native speakers, and in provinces characterized by elevated rates of private facility deliveries. Furthermore, the uptake of maternity incentives was lower in private health facilities and the Koshi, Bagmati, Madhesh, and Lumbini provinces.

Conclusions

The findings indicate that women from the most disadvantaged groups experience lower rates of routine maternal care visits and greater discontinuity of care from ANC to PNC, resulting in significant equity gaps compared to their advantaged counterparts. The rising trend of deliveries in private health facilities and cesarean sections, coupled with limited or nonexistent maternal incentives, imposes a financial burden on already marginalized populations. Health systems must prioritize designing and implementing targeted strategies tailored to local needs, acknowledging the significance of intersecting disadvantages and their influence on the equity and universality of maternal services in Nepal and other similar settings.

Keywords: Institutional delivery, cesarean section, maternal incentive, private health facilities, health equity, multiple marginalization.

Introduction

Over the past few decades, Nepal witnessed an increase in household income, wealth status, life expectancy, access to education, and basic health services (BHS)[1]. These improvements in socioeconomic status and access to services have enhanced health indicators. However, considerable disparities persist in wealth, access to better education, and health outcomes across the country. Wealth disparities significantly impact health equity by restricting access to health care services for individuals with lower income, resulting in poorer health outcomes [2].

Although overall, uptake of maternal health services has increased over the last two decades, progress among the most disadvantaged women groups is slow [3-5]. The reduction in maternal mortality ratio (MMR) per 100,000 live births, and neonatal mortality rate (NMR) per 1,000 live births has been slow and stagnant, with wide equity gaps between privileged and disadvantaged groups [6, 7]. The MMR decreased only from 281 to 259 nationally between 2006 and 2016 [22], while NMR has remained stagnant since 2016 [21]. Higher maternal deaths occurred among women with no or low levels of education [8]. Similar equity gaps existed in NMR, with the national average at 21, the lowest wealth quintile, and illiterate women having an NMR of 31, compared to 13 for the highest wealth quintile (NMR=13) [9].

The equity gaps extend beyond the maternal and newborn health (MNH) outcomes and are also reflected in the uptake of routine maternal health services in Nepal. The institutional delivery rate among the lowest wealth quintiles increased from 4 % to 66 % from 2006 to 2022; however, the growth is less impressive compared to the rise from 55% to 98% among the richest wealth quintiles during the same period [6, 9]. Moreover, the uptake of the institutional delivery among disadvantaged ethnic groups, such as Dalits (70%) and Madhesi (76%), is much lower than their privileged counterparts, such as Brahmin/Chhetri (87 %) [9]. Provincial inequities in health services are quite prevalent as well, with a lower institutional delivery rate among women living in Madhesh (67%) compared to women living in Sudurpashchim province (87%) in 2022 [9, 10]. Previous analysis also showed that the contact coverage of routine MNH visits was low among women with multiple forms of socioeconomic disadvantages (e.g., triple forms of disadvantage, i.e. women who were from the lowest wealth quintile and disadvantaged ethnicities and without education) compared to women with two or more forums of advantages [11].

The Government of Nepal (GON) has prioritized health equity in its policy and programs, aligning with global initiatives such as the Millennium Development Goals (2000–2015) and health-related Sustainable Development Goals (SDGs) by 2030 [12]. The National Health Policy 2019, aligned with constitutional mandates, aims to strengthen social health protection and ensure access to and use quality health services [13]. There have been emerging challenges such as increasing the private sector, where clients need to pay for the services that are available free of cost in the public sector, and inadequate provision of equitable and quality health services [14, 15].

Disparities also exist in the type and quality of health services available in private and public health facilities (HF) in Nepal. While access to free Basic Health Services (BHS) in the HF is the constitutional right of citizens [16], free-of-cost BHS are only available in public HF through government funding. In contrast, individuals must pay for services in private HF, even for BHS. Except for a few non-public hospitals enrolled on the Maternity scheme, which provides free maternal services, and the GON reimburses unit cost, such non-public hospitals are limited. Nevertheless, many prefer private HFs over public HFs due to the perceived better quality of care [17]. This preference persists despite the concerns about the profit motives driving over-medicalized care and poor regulation of accreditation, quality of care, and cost uniformity in private HFs [18].

Meanwhile, the readiness of public HF for MNH services remains suboptimal and inadequate compared to private HF[19]. Although the country has transitioned from a centralized to a federalized health system in recent years, introducing more locally relevant programs and a Gender Equality and Social Inclusion (GESI) approach for equitable policies and strategies, its implementation remains limited, leaving some population groups behind in accessing health services[20]. Additionally, the Aama program (maternity incentive program) has been implemented since 2005 to provide financial support as transportation costs for those who complete at least four ANC visits as per protocol and deliver babies at HFs[21-23]. This program is a promising initiative to improve maternal health in the country by incentivizing pregnant women to use HFs to give birth rather than giving birth at home and risking complications[21, 23]. In addition, the Aama program ensures free delivery services, normal, instrumental and CS, to women, while BHS only provides free normal delivery services[24, 25].

To achieve universal health coverage (UHC) and address health inequity more effectively, it is imperative to investigate the extent of inequities so that strategies and programs can be designed to ensure equitable access to MNH services for the populations most in need[22]. The importance of inequities is vital to formulate and revise policies, strategies, and programs in the context of the federalized health system of Nepal. Comprehensive evidence of trends in inequities and determinants could inform decision-makers in designing policies and implementing strategies for the most disadvantaged populations. Therefore, this study aims to investigate the equity gaps in key maternal health services, including institutional delivery, place of institutional delivery, delivery by cesarean section (CS), and uptake of maternal incentives while considering socioeconomic and demographic factors in Nepal. To gain a comprehensive understanding of the trends, this study specifically examines the overall trends of key maternal services in the three most recent NDHS from 2011, 2016, and 2022, while also investigating the levels and determinants of use of key maternal health services in the latest NDHS (2022).

Methods

Data sources

This study utilizes the publicly available NDHS data from 2011, 2016, and 2022. The data were obtained from the Demographic and Health Survey (DHS) Program (www.dhsprogram.com) with their approval and authorization. We conducted an overall national trends analysis of key maternal health services using data from three NDHS from 2011, 2016, and 2022. Determinants of key maternal health services were identified using data from the NDHS 2022. The sample included for trend analysis were women aged 15–49 years who had a live birth one year prior to NDHS 2011 (n=1,057), NDHS 2016 (n=964), and NDHS 2022 (n=981)[22]. Among 981 women, 796 (weighted) gave birth at HFs taken from NDHS 2022. In analysing determinants of types of institutional delivery, delivery by CS, and uptake of maternal incentives, the total sample size was 796 women.

Study variables

The outcome variables included institutional delivery, types of HF births (public or private HFs), delivery by CS, and uptake of maternal incentives. Independent variables included maternal age, religion, ethnicity, education, wealth quintile, marginalization status (disadvantages), province, place of residence, ecological region, occupation, native language, birth order of the child, place of delivery, and uptake of at least four ANC visits. Supplementary information (Table S1) includes the detailed categorization of each

independent variable. The marginalization status was created using three background variables: education, wealth status, and ethnicity [11, 26]. In the first step, the six ethnicities were categories into two groups according to their comparative privileges: disadvantaged ethnicities (including Dalit, Muslims, Terai caste, and disadvantaged Janajatis) and advantaged ethnicities (including Brahmin/Chhetri and advantaged Janajatis). Similarly, education status was dichotomized into illiterate (those who cannot read and write) and literate (those who can read and write and who have primary education or higher).
NDHS 2022 has five wealth quintiles, which were dichotomized into two groups, merging the lowest two quintiles as lower wealth status (lower 40%) and the upper three quintiles as upper wealth status (upper 60%). In the second step, by combining three variables with two categories, the study created a new variable (disadvantage status) with eight categories. We merged three categories with at least one form of disadvantage into one category, and the same was done for two forms of disadvantage in the next category. So, the new variable with disadvantage status has four categories: triple, double, single, and no disadvantages [11, 22].

Conceptual framework of study

The study adapted and modified the framework from Marmot [27, 28] and the WHO Social Determinants of Health framework [11] [Figure 1]. Individuals have structural factors (depending on the social structure of society and required political interventions) and intermediary factors (usually modifiable factors through multisectoral actions). These factors are intricately linked and overlapped with multiple identities, thereby creating intersectional (dis)advantages. These factors influence the access to and use of maternal health services. Based on this framework, the study selected some independent variables: intersectional disadvantages, structural factors (religion, ethnicity, wealth status, and occupation), and intermediary factors (maternal age, province, ecological region, place of residence, birth order, native language, and uptake of at least four ANC visits).

……Insert figure here …………….

Figure 1: conceptual framework to guide the analysis

Data analyses

We performed univariate, bivariate, and multivariate analyses. We analyzed proportions for each outcome variable, examining the association with all independent variables and assessing differences in proportions using chi-square tests. Before running the final regression model, the study checked multicollinearity and excluded independent variables with variation inflation factor (VIF) ≥5. The variable on disadvantage status was created

using wealth status, ethnicity, and education. The study reported the adjusted odds ratios (AOR) with a 95 % confidence interval (CI), and the statistical significance level was set at $p<0.05$ (two-tailed). The study reported weighted estimates and conducted all analyses using the “svy” command function and considering the clustering effect in Stata 17 (Stata Corp, 2023).

Research ethics

We used publicly accessible, de-identified datasets from the Demographic and Health Survey program (https://dhsprogram.com/data/available-datasets.cfm). For each round of NDHS, the survey received ethical approval from the ICF Institutional Review Board in the USA and the Nepal Health Research Council, Nepal. Mothers provided informed consent both for themselves and their children during those surveys. Given that this research involved only a secondary analysis of completely anonymised data, there was no need for additional ethical clearance. The author (RBK) received approval to access and utilize the de-identified data.

Results

Trends of ANC, Institutional Delivery and PNC (2011 to 2022)

Figure 2 presents trends in at least 4ANC visits, institutional delivery and PNC within the first 48 hours of childbirth by marginalization status. At the national level, the overall trends were increasing for all routine maternal services from 2011 to 2022 for all dis(advantage) groups. For instance, 4ANC uptake among the triple disadvantage group increased from 27% in 2011 to 50% in 2016 and to 67% in 2022. However, the uptake of each type of maternal service showed a decline from 4ANC to PNC for all (dis)advantage groups. For instance, in the NDHS 2022, the triple disadvantage group had a 4ANC rate of 82%, an institutional delivery rate of 81%, and a PNC rate of 73%, resulting in full continuum care at 59%. Uptake of routine maternal health care services was highest among women with no disadvantages and lowest among women with triple disadvantages. The equity gap between all four marginalization status categories declined over time, illustrated by the converging trendlines. From 2011 to 2022, there was a statistically significant increase in the uptake of at least 4ANC visits for all women with at least one disadvantage (66% in 2011, 75% in 2016, and 86% in 2022). The increase in institutional deliveries and PNC visits from 2011 to 2022 was statistically significant for all categories of marginalization status. The proportion of women who have completed all three routine maternal care visits (4ANC, HF delivery and PNC) increased significantly from 2011 to 2022 for all women with at least one disadvantage.

……Insert figure 2 here …………….

Figure 2: trends of uptake of routine maternal health services among women who had a live birth one year prior to the survey in the three most recent surveys, by marginalization status.

Trends of place of delivery

Figure 3 illustrates the trend of place of delivery over the three NDHS. The childbirth trends at both public and private HFs from 2011 to 2022. For instance, childbirth at public HFs increased from 36% to 63% from 2011 to 2022, while at private HFs, it increased from 11% to 18% in the same duration. Despite the decline in home delivery without assistance over the years, they still accounted for 19% of all women who gave birth, as reported in NDHS 2022.

……Insert figure 3 here …………….

Figure 3: Trends of place of delivery over three NDHS surveys among women who had live birth one year prior to the survey in the three most recent surveys.

Trends of delivery by cs and uptake of maternal incentive

Figure 4 illustrates the trends of delivery by CS and the uptake of maternal incentives among women who had at least a live birth and gave birth in a HF. The national trend for all women in CS rates nearly doubled from 13% in 2011 to 25% in 2022. Delivery by CS was generally highest among women, with no disadvantage or a single disadvantage. Among those women who gave birth in HFs (both public and private), the uptake of maternal incentives decreased from 73% in 2011 to 67% in 2022.

……Insert figure 4…… here …………….

Figure 4: Trends of delivery by CS and uptake of maternal incentive among women who had at least a live birth and gave birth in HFs one year prior to the survey in the three most recent surveys by marginalization status.

Trends of delivery by CS in public and private HF

Figure 5 shows the trend of delivery by CS in public and private HFs in the three NDHSs. From 2011 to 2022, the rate of delivery by CS in public HFs increased from 9 % to 18%, while in private HFs, it increased from 30 % to 51 % in the same period.

……Insert figure 5…… here …………….

Figure 5: trends of uptake of delivery by CS among women who had at least a live birth and gave birth in HFs one year prior to the three most recent surveys.

Trends of the uptake of maternal incentives in public and private HFs

Figure 6 presents the uptake of maternal incentives by public and private HFs (not all private HFs had the Aama program) births in the three NDHS. Among women who gave birth in a public HF, the uptake of maternal incentives showed a declining trend. Nearly 9 in 10 (87%) women received maternal incentives in 2011, which declined to 78 % in 2022. Among those who gave birth in private HFs, the proportion of women receiving maternal incentives has remained low at 29% in 2011 and 2022, slightly increasing to 40% in 2016.

……Insert figure 6…… here …………….

Figure 6: trends of the uptake of maternal incentives among women who gave birth in HFs in the three most recent surveys.

Institutional delivery among women who had live birth one year prior to the NDHS 2022

Table 1 presents the background characteristics of the study participants. In NDHS 2022, a high proportion of women were in the 20–24-year age group (36.8%), from Janajati backgrounds (31.9%), with secondary education (44.9%), from Madhesh province (24.4%), with their first birth (40.7%), and with at least one form of disadvantage (43.4%).

Table 1: Distribution of women aged 15–49 years who had a live birth one year prior to the survey by background variables, NDHS 2022.

**Maternal characteristics**	**%**	**95 % CI**	**N=981**
**Structural factors**			
**Religion**			
Hindu	83.7	79.8–87	822
Other	16.3	13.0–20.2	160
**Ethnicity**			
Brahmin	8.3	6.4–10.8	81
Chhetri	18.7	15.6–22.1	183
Madheshi	17.0	13.7–21.0	167
Dalit	17.2	13.9–21.1	169
Janajati	31.9	27.8–36.3	313
Muslim	7.0	4.4–10.9	68
**Education**			
No education	16.4	13.6–19.7	161
Basic	33.4	30.2–36.8	328
Secondary	44.9	40.8–49.1	441
Higher	5.2	3.3–8.1	51
**Wealth quintile**			
Poorest	20.4	17.4–23.7	200
Poorer	21.6	18.6–25.1	212
Middle	20.1	17.1–23.5	197
Richer	20.2	17.0–23.7	198
Richest	17.7	14.4–21.6	174
**Occupation**			

**Maternal characteristics**	**%**	**95 % CI**	**N=981**
Not working	44.9	41.1–48.7	440
Agriculture	40.6	36.6–44.7	398
Manual labor	5.8	4.3–7.9	57
Working paid	8.7	6.9–11.0	86
**Marginalization status**			
**Disadvantages**			
Triple	8.3	6.5–10.6	82
Double	29.8	26.3–33.7	293
Single	43.4	39.5–47.4	426
No	18.4	15.0–22.4	180
**Intermediary factors**			
**Province**			
Koshi	19.2	16.3–22.4	188
Madhesh	24.4	21.3–27.7	239
Bagmati	16.3	13.2–19.9	160
Gandaki	6.7	5.1–8.7	66
Lumbini	15.9	13.8–18.2	156
Karnali	7.1	6.1–8.2	70
Sudurpashchim	10.4	9.0–12.0	102
**Residence**			
Urban	66.0	62.8–69.2	648
Rural	34.0	30.8–37.2	333
**Ecoregion**			
Mountain	6.2	3.9–9.5	60
Hill	34.9	30.0–40.0	342
Terai	59.0	53.8–64	579
**Native language**			
Nepali	48.5	43.9–53.2	476
Maithili	18.8	14.5–23.9	184
Bhojpuri	8.5	5.5–12.8	83
Other	24.2	20.6–28.2	237

Maternal characteristics	%	95 % CI	N=981
**Age in Years**			
< 20	18.8	16.4–21.6	185
20–24	36.8	33.8–39.9	361
25–29	27.6	24.6–30.9	271
30–34	12.7	10.2–15.9	125
35 and above	4.0	2.8–5.6	39
**Birth order**			
1	40.7	36.9–44.6	400
2	36.8	33.3–40.5	361
3+	22.4	19.5–25.7	220
**Place of Delivery**			
Public HF	63.5	59.3–67.6	623
Private HF	17.6	14.3–21.4	172
Home	18.9	16.0–22.2	185

Descriptive analysis of institutional delivery, delivery in private HF, delivery by CS, and uptake of maternal incentives, NDHS 2022

Among 981 study participants, 796 (81.1%) delivered their babies at HF. Nine background variables were significantly associated with institutional delivery (Figure 10). Large equity gaps existed between Brahmins (90.8%) and Muslims (68.7%), women with no education (64.3%) and those with higher education (100%), women in the highest wealth quintile (96.7%) and those in the lowest (71.6%), women with triple disadvantages (60.2%) and women with no disadvantages (96.6%), women in Madhesh province (64.8%) and those in Bagmati province (94.4%), Maithili speakers (64.1%) and Nepali speakers (91.4%), and women with a third or higher birth order (63.5%) and those with a first birth order (91.3%). See Supplementary Information Table S2 for findings on additional background variables.

Among all institutional deliveries (n=796), more than one in five women (21.6%) gave birth in private HF. Seven background variables were significantly associated with delivery in a private HF. Compared with the national average and reference categories for each variable, delivery at private HF was high among Muslim women (41.2%), women with higher education (42.5%), women in the highest wealth quintile (31.8%), women from Koshi province (35.7%), women from the Terai ecoregion (27.4%), women with paid jobs (29.4%), and Maithili speakers

(38.5%). See Supplementary Information Table S3 for findings on additional background variables.

Among all institutional deliveries (n=796), one in four women (25%) had delivery by CS. Compared with the national average and the other categories for each variable, delivery by CS was more common among women aged 35 and older (39.5%), women with higher education (42.6%), women in the highest wealth quintile (41.6%), women with no disadvantages (38%), women in Bagmati province (37.9%), women with paid jobs (39.3%), and women with a second birth order (33.1%). See Supplementary Information Table S4 for findings on additional background variables.

Of 796 women who gave birth in HF, two-thirds (67.3%) received maternal incentives. Compared with the national average and respective reference categories for each variable, uptake of maternal incentives was higher if women were of Chhettri ethnicity (73.8%), from the lowest wealth quintile (79.3%), from Gandaki province (90.4%), from the Mountain ecoregion (83.5%), or spoke other languages (74.1%). See Supplementary Information Table S5 for findings on additional background variables.

Determinants of institutional delivery

Supplementary information Table S6 presents the crude odds ratio (COR) for background variables associated with delivery in HF in the 1 year prior to the 2022 NDHS. Table 2 presents the adjusted odds ratio (AOR) and their 95% confidence intervals (CI) for various background characteristics associated with institutional delivery in 2022 NDHS. Lower odds of institutional delivery were observed for women under 20 years old (AOR=0.50; 95% CI:0.28–0.88) and those from Karnali (AOR=0.34; 95% CI:0.15–0.76) when compared to women aged 20–24 years and residing in Sudurpashchim [Table 2]. Similarly, lower odds of institutional delivery were found among women with triple (AOR=0.29; 95% CI:0.09–0.9) and double (AOR=0.29; 95% CI:0.11–0.79) forms of disadvantages as compared to women with no disadvantage. Institutional delivery was lower among women working in the agricultural sector (AOR=0.61; 95% CI:0.39–0.97) as compared to those not working. Furthermore, institutional delivery was lower among women who are native Maithili speakers (AOR=0.38; 95% CI:0.16–0.91), Bhojpuri speakers (AOR=0.35; 95% CI:0.13–0.92) and other language speakers (AOR=0.41; 95% CI:0.22–0.77), as compared to women who are native Nepali speakers. Compared to women having their first birth, those with a second birth had lower odds of institutional delivery (AOR=0.24; 95% CI:0.13–0.43), while women with a third of higher birth order had even lower odds (AOR=0.16; 95% CI:0.08–0.30). On the

other hand, higher odds of institutional delivery were found if women had completed at least four ANC visits (AOR=1.78; 95% CI:1.18–2.68).

Table 2: determinants of institutional delivery for women who had a live birth one year before the survey, NDHS 2022.

**Maternal characteristics**	**Categories**	**AOR**	**95% CI**
**Occupation**	Not working	1.00	
	Agriculture	0.61*	0.39–0.97
	Manual labor	0.82	0.32–2.08
	Working paid	1.11	0.39–3.20
**Religion**	Hindu	1.00	
	Other than Hindu	1.04	0.59–1.84
**Province**	Koshi	0.66	0.32–1.38
	Madhesh	0.42	0.16–1.10
	Bagmati	1.11	0.41–3.01
	Gandaki	0.63	0.25–1.59
	Lumbini	0.65	0.27–1.54
	Karnali	0.34**	0.15–0.76
	Sudurpashchim	1.00	
**Disadvantages**	Triple	0.29*	0.09–0.90
	Double	0.29*	0.11–0.79
	Single	0.51	0.18–1.40
	No	1.00	
**Residence**	Urban	1.00	
	Rural	0.92	0.61–1.40
**Native language**	Nepali	1.00	
	Maithili	0.38*	0.16–0.91
	Bhojpuri	0.35*	0.13–0.92
	Other	0.41**	0.22–0.77
**Age**	<20	0.50*	0.28–0.88
	20–24	1.00	
	25–29	0.98	0.60–1.60
	30–34	1.47	0.71–3.04

	35 and above	1.26	0.52–3.02
**Birth order**	First	1.00	
	Second	0.24***	0.13–0.43
	Third or Higher	0.16***	0.08–0.30
**At least four ANC visits**	No	1.00	
	Yes	1.78**	1.18–2.68

Note: * p<0.05, ** p<0.01, *** p<0.001.

Determinants of institutional delivery in private HF

Supplementary information Table S7 presents the CORs for background variables associated with delivery in private HF in the 1 year prior to the 2022 NDHS. Table 3 demonstrates the determinants of live childbirth in private HF. As compared to Sudurpashchim, the odds of delivery in private HF were higher if women were from Koshi (AOR=6.53; 95% CI:2.71–15.74), Madhesh (AOR=4.69; 95% CI:1.6–13.75), Bagmati (AOR=2.71; 95% CI:1.02–7.19), and Lumbini (AOR=2.69; 95% CI:1.13–6.4) provinces. Furthermore, women with a single form of disadvantage (AOR=3.68; 95% CI:1.40–9.63) and no forms of disadvantages (AOR=3.7; 95% CI:1.18–11.6) had higher odds of delivery in private HF compared to those with triple forms of disadvantages. On the other hand, there were lower odds of childbirth in private HF if women were from Karnali province (AOR=0.08; 95% CI:0.01–0.63), working in manual labor (AOR=0.35; 95% CI:0.13–0.94), and birth order three or more (AOR=0.43; 95% CI:0.19–0.95) compared to women residing in Sudurpashchim province, women without any work, and women with first birth order [Table 3].

Table 3: determinants of childbirth in private HF among women who had a live birth one year before the survey, NDHS 2022.

Maternal characteristics	Categories	AOR	95% CI
**Occupation**	Not working	1.00	
	Agriculture	0.95	0.58–1.55
	Manual labor	0.35*	0.13–0.94
	Working paid	1.23	0.55–2.76
**Religion**	Hindu	1.00	
	Other than Hindu	0.99	0.56–1.75
**Disadvantages**	Triple	1.00	
	Double	1.76	0.64–4.88
	Single	3.68**	1.40–9.63

	No	3.70*	1.18–11.6
**Province**	Koshi	6.53***	2.71–15.74
	Madhesh	4.69**	1.60–13.75
	Bagmati	2.71*	1.02–7.19
	Gandaki	0.91	0.28–2.97
	Lumbini	2.69*	1.13–6.40
	Karnali	0.08*	0.01–0.63
	Sudurpashchim	1.00	
**Residence**	Urban	1.00	
	Rural	0.97	0.59–1.59
**Native language**	Nepali	1.00	
	Maithili	2.10	0.83–5.34
	Bhojpuri	0.78	0.25–2.44
	Other	0.87	0.49–1.54
**Age**	<20	1.00	
	20–24	0.82	0.43–1.58
	25–29	1.15	0.54–2.47
	30–34	2.12	0.82–5.50
	35 and above	0.76	0.21–2.75
**Birth order**	First	1.00	
	Second	0.69	0.40–1.19
	Third or higher	0.43*	0.19–0.95
**At least four ANC visits**	No	1.00	
	Yes	0.98	0.52–1.88

Note: * p<0.05, ** p<0.01, *** p<0.001.

Determinants of delivery by CS

Supplementary information Table S8 presents the CORs for background variables associated with delivery by CS in the 1 year prior to the 2022 NDHS. Table 4 demonstrates the determinants of CS among women who had live births one year before the survey from NDHS 2022. The study found higher odds of delivery by CS if women were aged 25–29 years (AOR=4.42; 95% CI:2.05–9.54), 30–34 years (AOR=4.57; 95% CI:1.84–11.31), and 35 years and above (AOR=5.67; 95% CI:1.7–18.94) compared to women who were teenage

mothers. Similarly, this study found higher odds of delivery by CS if women were from Koshi (AOR=4.53; 95% CI:2.26–9.11), Madhesh (AOR=2.76; 95% CI:1.17–6.5), Bagmati (AOR=3.45; 95% CI:1.59–7.52), and Lumbini (AOR=2.49; 95% CI:1.19–5.22) provinces compared to women from Sudurpashchim province. Higher delivery by CS was also found among native Nepali speakers (AOR=1.91; 95% CI:1.04–3.5) and native Maithili speakers (AOR=2.13; 95% CI:1.05–4.32) compared to women who speak other languages. Lower odds of delivery by CS were found among women working in the agricultural sector (AOR=0.48; 95% CI:0.30–0.79) compared with nonworking women. Additionally, women with a birth order of three or more had lower (AOR=0.4; 95% CI:0.19–0.84) odds of delivery by CS compared to women with a first birth order [Table 4].

Table 4: determinant of delivery by cs for women who had a live birth in HF one year prior to the survey, NDHS 2022.

**Maternal characteristics**	**Categories**	**AOR**	**95% CI**
**Occupation**	Not working	1.00	
	Agriculture	0.48**	0.30–0.79
	Manual labor	0.64	0.24–1.75
	Working paid	0.87	0.43–1.78
**Religion**	Hindu	1.00	
	Other	0.96	0.50–1.84
**Disadvantages**	Triple	1.00	
	Double	1.62	0.54–4.87
	Single	2.14	0.77–5.91
	No	1.83	0.64–5.2
**Province**	Koshi	4.53***	2.26–9.11
	Madhesh	2.76*	1.17–6.50
	Bagmati	3.45**	1.59–7.52
	Gandaki	1.88	0.76–4.66
	Lumbini	2.49*	1.19–5.22
	Karnali	0.91	0.33–2.51
	Sudurpashchim	1.00	
**Residence**	Urban	1.00	
	Rural	0.71	0.46–1.10
**Native language**	Nepali	1.91*	1.04–3.50

Maternal characteristics	Categories	AOR	95% CI
	Maithili	2.13*	1.05–4.32
	Bhojpuri	0.82	0.24–2.87
	Others	1.00	
**Age**	<20	1.00	
	20–24	1.99	0.99–3.99
	25–29	4.42***	2.05–9.54
	30–34	4.57**	1.84–11.31
	35 and above	5.67**	1.7–18.94
**Birth order**	First	1.00	
	Second	1.04	0.64–1.69
	Third or Higher	0.4*	0.19–0.84
**At least four ANC visits**	No	1.00	
	Yes	1.11	0.55–2.26

Note: * p<0.05, ** p<0.01, *** p<0.001.

Determinants of the uptake of maternal incentives

Supplementary information Table S9 presents the CORs for background variables associated with the uptake of maternal incentives in the 1 year prior to the 2022 NDHS. Table 5 demonstrates the determinants of uptake of maternal incentives among women in NDHS 2022. The analysis found lower odds of uptake of maternal incentives among women from Koshi (AOR=0.11, 95% CI:0.05–0.25), Madhesh (AOR=0.18, 95% CI:0.13–0.7), Bagmati (AOR=0.3, 95% CI:0.13–0.7), and Lumbini (AOR=0.33, 95% CI:0.16–0.71) provinces compared to Sudurpashchim province.

Table 5: determinants of uptake of maternal incentives by women who had a live birth one year prior to the survey, NDHS 2022.

Maternal characteristics	Categories	AOR	95% CI
**Occupation**	Not working	1.00	
	Agriculture	1.08	0.71–1.64
	Manual labor	1.65	0.75–3.63
	Working paid	0.8	0.45–1.42
**Religion**	Hindu	1.00	
	Other	1.75	0.94–3.26
**Disadvantages**	Triple	1.00	

Maternal characteristics	Categories	AOR	95% CI
	Double	1.03	0.45–2.35
	Single	0.58	0.26–1.31
	No	0.74	0.29–1.91
**Province**	Koshi	0.11***	0.05–0.25
	Madhesh	0.18***	0.07–0.45
	Bagmati	0.3**	0.13–0.70
	Gandaki	1.54	0.49–4.82
	Lumbini	0.33**	0.16–0.71
	Karnali	0.86	0.40–1.86
	Sudurpashchim	1.00	
**Residence**	Urban	1.00	
	Rural	1.09	0.71–1.66
**Native language**	Nepali	0.75	0.44–1.28
	Maithili	0.51	0.24–1.06
	Bhojpuri	0.65	0.24–1.73
	Other	1.00	
**Age**	<20	1.00	
	20–24	1.20	0.71–2.04
	25–29	1.22	0.65–2.30
	30–34	0.69	0.29–1.66
	35 and above	1.05	0.36–3.05
**Birth order**	First	1.00	
	Second	1.46	0.91–2.34
	Third or higher	1.61	0.84–3.09
**At least four ANC visits**	No	1.00	
	Yes	1.37	0.81–2.29

Note: ** p<0.01, *** p<0.001.

Discussion

This study reveals significant inequities across various demographics, indicating lower completion rates and high discontinuation along the maternal continuum, particularly among women facing multiple forms of disadvantage compared to their advantaged counterparts in Nepal. It highlights disparities in institutional delivery rates, with lower rates observed among women from Karnali and Madhesh provinces, native speakers of Terai languages (Maithili and Bhojpuri), and those with higher birth orders. Conversely, the completion of at least four ANC visits is associated with higher rates of institutional delivery, underscoring the importance of early and consistent maternal care. The trend toward CS has increased in the private HF, especially among women with fewer disadvantages and older age groups. In contrast, women engaged in manual labor, and those with higher birth orders were less likely to utilize private health services. The utilization of private health services was notably higher in the Koshi, Bagmati, Madhesh, and Lumbini provinces. Additionally, the uptake of maternal incentives was significantly lower among those who opted for private health services and underwent delivery by CS (not all private HFs implement maternity schemes). These findings underscore critical inequities in maternal health, emphasizing the necessity for targeted interventions and a layered approach that addresses the trends and key determinants affecting access to maternal care for disadvantaged women.

Low continuum of care and wide equity gaps among intersectional groups

This study found a high catch-up rate for maternal care along the continuum of care; however, it shows a low keep-up rate with high dropouts occurring around childbirth and the first PNC visit. The completion rates of all three routine maternal care visits were low, with a higher uptake of ANC observed in early gestational months, followed by a significant decline in the later weeks of pregnancy. Difficulties in reaching HF as women near childbirth, coupled with a lack of transportation and insufficient availability of uninterrupted care across the continuum, could have contributed to the imbalance between catch-up and keep-up rates [29]. The combination of high institutional delivery and low PNC reveals that many mothers who gave birth in HF did not receive PNC. These findings were consistent with the further analysis of NDHS 2016 [30] and similar studies conducted in other low- and middle-income countries, such as Zambia [31] and Pakistan [32]. The higher rates of first ANC visits, followed by drops during the late gestational age and childbirth, could be attributed to physical barriers to accessing HF, particularly due to distance and inadequate transportation facilities. For instance, most HFs in Nepal are in the center of municipalities, which might be

inaccessible for some remote communities under their catchment areas. Improving transportation facilities could enhance access to HF during the gestational weeks around childbirth [33, 34]. Another solution could be establishing waiting homes for women from remote areas to minimize the long travel time for women nearing childbirth. Evidence suggests maternal waiting homes could improve institutional delivery and perinatal health outcomes [35]. Ensuring adherence to quality of care guidelines could facilitate the continuum of care from ANC and PNC, and mandating PNC before discharge from HF after delivery could prove beneficial [30]. Moreover, the Ministry of Health and Population (MoHP) could implement multiple operational and strategic (and long-term) approaches to improve routine maternal care visits [36].

Low institutional delivery among socioeconomically and geographically defined disadvantaged groups

This study identified an overall increase in institutional delivery rates. However, it also revealed lower odds of institutional delivery among women with multiple forms of disadvantages, such as poverty and lack of education, those residing in remote provinces like Karnali, and women with higher birth orders. Women with multiple forms of disadvantages face structural inequities, have difficulty in living conditions [37], and could face several access barriers to reach HF [38], including difficult geographic terrains and distance to HF. Despite the implementation of the Aama program that was designed to address financial barriers, expand birthing centers to address geographic, and train SBAs to improve the access and quality of childbirth services in birthing centers. However, evidence suggests that care quality at birthing centers needs to improve [17, 38]. This study also showed that native regional language speakers, particularly in the Terai regions, such as Maithili and Bhojpuri, had lower odds of delivery in HF compared to native Nepali speakers. The cultural upbringings and belief systems related to care-seeking practices and the sharing of reproductive and maternal health issues could have influenced the disparities. The language barriers between the Nepali-speaking care providers and those who cannot speak Nepali may hinder effective communication of health needs, potentially widening equity gaps. Health care providers who share similar ethnic, cultural, and linguistic backgrounds with local women have a better opportunity to effectively address their maternal health issues [39].

Increasing institutional delivery in private HF

This study showed an increasing trend of delivery in private HF with higher odds of childbirth in provinces with better socioeconomic development indicators, such as Koshi and

Bagmati, and provinces that cover the Terai region, such as Madhesh and Lumbini, among relatively privileged women with single or no disadvantages. In principle, private health services complement UHC as their role is vital, whereas the public health system is inadequate in providing health services to its population. However, in the context of the poorly regulated private health sector and the lack of a health insurance program, private health services can financially burden people and push them to further marginalization. Earlier evidence suggests that bypassing local HF to go to bigger hospitals and public hospitals in nearby cities caused overcrowding that further compromised the quality of maternal care[40, 41]. People sought private care to receive better quality of care, despite having to pay for those services [41]. Private HFs that are profit motivated often intent on over-prescription of unnecessary tests, medications, and surgeries, which also increase the burden of care cost, especially for socioeconomically disadvantaged groups[42, 43]. Nepal's private health services are fee-for-service, and people must pay for health services. The GON has implemented an Aama program in all public HFs, but very few private HFs have implemented this program [44]. However, the care-seeking patterns for childbirth in private HF are increasing. Seeking care in private HF means paying for services that are freely available in public HF [42].

High and increasing trends of delivery by CS

This study found that delivery by CS in private HF is increasing. High delivery by CS was found if women were pregnant at an older age in Koshi, Bagmati, Lumbini, or Madhesh provinces and among Maithili native-speaking women. The WHO has recommended that 10–15 % of childbirth should require delivery by CS[45]. However, Nepal has a higher rate of CS than the WHO recommended in general and is three times higher if women were in private HF.[46] More than half of the total deliveries at private HF are by CS. Among women who had multiple forms of disadvantages (poor, illiterate, and without education), more than two-thirds (72 %) had childbirth in private HF by CS. Women from Koshi, Bagmati, Lumbini, and Madhesh provinces had higher odds of childbirth by CS in private HF. Madhesh province, where most Maithili-speaking women live, had high delivery by CS in private HF. Madhesh province has lower socioeconomic indicators, including female literacy and distinct linguistic and cultural diversity, which influence the ability to reach HF for services, especially in pregnancy and childbirth [47]. Higher CS rates in private HF may be linked with revenue-generation mechanisms for both public and private HF and providers rather than being medically recommended. Medicalization and commercialization of routine

care services can have high-cost implications for those socioeconomically disadvantaged populations. In public hospitals too, the HFs get higher unit cost reimbursement for CS through maternity incentive program (CS- NRs 10,000 equivalent to 90 USD). The monitoring system needs to be strengthened to ensure the CS services are provided only when medically indicated.

Low and decreasing trends in uptake of maternal incentives

Overall, less than two-thirds of all women who gave birth in HF received maternal incentives nationally, and the trend is decreasing. The proportion of women with multiple forms of marginalization who received maternal incentives was higher than their privileged counterparts. Nearly four in five women received incentives if they delivered babies in public HF, while the uptake of incentives was notably decreasing among women who gave birth in private HF. This could be because most public HFs have implemented the Aama program, while not all private HFs have adopted it. This could also have contributed to the lower uptake rates in private HFs than public HFs [21]. Provinces with higher rates of childbirth in private HF (Koshi, Bagmati, Madhesh, and Lumbini) and higher rates of delivery by CS had lower odds of uptake of maternal incentives. Families continue to make out-of-pocket expenses for birth in private HFs, despite receiving maternal incentives in public hospitals.[48] Concerns about quality of care, high delivery in the private sector, and high CS delivery costs in private HFs, along with the lack of uniformity of maternal incentives program between private and public HF should be reevaluated to align with the changing context of the decentralized health system in Nepal.

Program and policy implications

Some policy and program implications can be drawn from this study. First, policies should focus on delivering health services to the most disadvantaged groups by addressing structural and intersectional disadvantages, improving conditions where they live, and enhancing access to quality healthcare through multisectoral collaboration. Second, to boost completion rates for maternal care, the maternal incentive program should be integrated with continuity of care indicators and tracked in the health management information system (HMIS). Policies must also ensure that women receive at least one PNC visit within 24 hours of childbirth for mothers and newborns to secure incentives. Third, healthcare providers need to follow mandatory PNC for mothers and newborns policies for mothers delivering in HF, supported by regular training and quality monitoring. Improved communication during ANC visits

about childbirth locations is essential. Fourth, recruiting local healthcare providers can enhance communication and care in provinces like Madhesh and Lumbini, which have high populations of Maithili- and Bhojpuri-speaking people. Additionally, improving transportation in Karnali province is crucial to increasing access to HF for childbirth. Fifth, ensuring uninterrupted access to quality childbirth services in HF can enhance trust in government services. Local healthcare providers can better identify women facing multiple disadvantages and tailor service delivery strategies accordingly. It's essential to regulate the private sector by enforcing guidelines, such as allocating 10% of beds for marginalized populations and standardizing care costs. The government should also promote the Aama program in private HF to create a supportive environment. As care-seeking in private HF rises due to a lack of trust in public facilities, improving quality in public HF, particularly in remote areas and Madhesh province, is crucial. The Aama program must be effectively implemented in both public and private settings. Finally, the rising rate of CS in the private sector is concerning, as it's unclear if these procedures are medically necessary or requested by patients. Effective health information and client education on the benefits and risks of normal delivery and CS are essential. Incentives through maternity programs should be revised to specifically target women of multiple marginalization, such as poor women who belong to disadvantaged ethnicities and those who live in remote areas. Engaging local leaders and stakeholders in understanding the impact of intersecting disadvantages can strengthen health policy efforts at both local and provincial levels.

Study limitations

The study included the mothers who had live births in the one year prior to the surveys. The study used this short window of time to reduce the recall bias of childbirth-related information and identify the recent history of maternal care. However, this short window of time has the implications of a small sample size in some of the categories of variables. Therefore, the study merged some categories of variables (e.g., marginalization status) with a small sample size. Additionally, the study created disadvantages using three variables—education, wealth status, and ethnicity—which were multicollinear with the multiple marginalization status. Thus, the study has excluded these three variables in the regression analysis. The analysis of the uptake of maternal incentives in private HFs should be interpreted carefully since the denominator includes all private HFs, regardless of whether they are implementing the maternal incentive scheme.

Conclusions

This study finds that while first ANC visits and institutional births are high, PNC visits and completion of all three maternal care visits are low, with significant equity gaps. Women who are facing multiple disadvantages, and those from rural areas have lower access to maternal care compared to more privileged groups. There is a notable rise in childbirth and delivery by CS in private HFs, particularly among Maithili- and Bhojpuri-speaking women and those from specific provinces, but low uptake of maternal incentives. The study recommends focused efforts to improve completion rates from ANC to PNC, better data integration into health monitoring systems, and regular training for care providers to enhance service quality. The Aama program should be expanded to cover the full continuum of care uniformly across facilities. Addressing supply-side barriers, recruiting local health workers, and improving public trust in health systems are essential, especially in remote communities. Additionally, auditing CS deliveries for medical necessity and engaging local leaders to address health inequities are crucial. Further qualitative research is needed to explore the uptake of maternal health services among women of disadvantaged groups.

Acknowledgements

The authors would like to thank the DHS program for granting permission to use the data for further analysis.

References

1. Ministry of Health and Population: **Department of Health Services (DoHS) Annual Report 2023-24** In. Department of Health Services. Kathmandu, Nepal.: Ministry of Health and Population 2024.
2. Ward JL, Viner RM: **The impact of income inequality and national wealth on child and adolescent mortality in low and middle-income countries**. *BMC Public Health* 2017, **17**(1):429.
3. Langlois EV, Miszkurka M, Zunzunegui MV, Ghaffar A, Ziegler D, Karp I: **Inequities in postnatal care in low- and middle-income countries: a systematic review and meta-analysis**. *Bull World Health Organ* 2015, **93**(4):259-270G.
4. Malqvist M, Pun A, Raaijmakers H, Kc A: **Persistent inequity in maternal health care utilization in Nepal despite impressive overall gains**. *Global Health Action* 2017, **10**(1):1356083.
5. Countdown to C: **Countdown to 2030: tracking progress towards universal coverage for reproductive, maternal, newborn, and child health**. *Lancet* 2018, **391**(10129):1538-1548.
6. Ministry of Health and Population, New ERA, ICF International Inc: **Nepal demographic and health survey 2006**. In. Kathmandu,Nepal :Ministry of Health and Population, New ERA, and ICF International, Calverton, Maryland; 2007.
7. Ministry of Health and Population, New ERA, ICF International Inc: **Nepal demographic and health survey 2016.** In. Kathmandu,Nepal :Ministry of Health and Population, New ERA, and ICF International, Calverton, Maryland; 2017.
8. Ministry of Health and Population; National Statistics Office: **National Population and Housing Census 2021: Nepal Maternal Mortality Study 2021.** . In. Kathmandu: Ministry of Health and Population; National Statistics Office; 2022.
9. Ministry of Health and Population, New ERA, ICF International Inc: **Nepal Demographic and Health Survey 2022** In. Kathmandu,Nepal :Ministry of Health and Population, New ERA, and ICF International, Calverton, Maryland; 2022.
10. World Health Organisation: **Health equity assessment toolkit.** In. Geneva: World Health Organisation, ; 2020.
11. Khatri RB, Alemu Y, Protani MM, Karkee R, Durham J: **Intersectional (in) equities in contact coverage of maternal and newborn health services in Nepal: insights from a nationwide cross-sectional household survey**. *BMC Public Health* 2021, **21**(1):1098.
12. NPC: **National Planning Commission, 2015: Sustainable Development Goals, 2016-2030, National Report. Government of Nepal, National Planning Commission, Kathmandu, Nepal** In.; 2015.
13. Ministry of Health and Population: **National health policy 2019**. In. Kathmandu; 2019.
14. Khatri RB, Assefa Y, Durham J: **Multidomain and multilevel strategies to improve equity in maternal and newborn health services in Nepal: perspectives of health managers and policymakers**. *Int J Equity Health* 2023, **22**(1):105.
15. Wasti SP, van Teijlingen E, Rushton S, Subedi M, Simkhada P, Balen J, Nepal Federal Health System T: **Overcoming the challenges facing Nepal's health system during federalisation: an analysis of health system building blocks**. *Health Res Policy Syst* 2023, **21**(1):117.
16. Constituent Assembly Secretariat: **Constitution of Nepal 2015.** In. Kathmandu: Constituent Assembly Secretariat; 2015.
17. Karkee R, Lee AH, Pokharel PK: **Women's perception of quality of maternity services: a longitudinal survey in Nepal**. *BMC Pregnancy Childbirth* 2014, **14**(1):45.
18. Prakash S: **Increasing trends in unhealthy practices of clinical laboratory medicine service in Nepal**. *Janaki Medical College Journal of Medical Science* 2017, **5**(1):33-48.

19. Tuladhar S, Paudel D, Rehfuess E, Siebeck M, Oberhauser C, Delius M: **Changes in health facility readiness for obstetric and neonatal care services in Nepal: an analysis of cross-sectional health facility survey data in 2015 and 2021**. *BMC Pregnancy Childbirth* 2024, **24**(1):79.
20. Ministry of Health and Population: **Health Sector Gender Equality and Social Inclusion Strategy** In.: Ministry of Health, Department of Health Services. Kathmandu, Nepal.; 2020.
21. Ministry of Health and Population, NHSSP: **Review of the maternity incentive and free delivery care programme (the Aama Suraksha programme) in Nepal**. In. Kathmandu, Nepal 2020.
22. Khatri R, Dulal KP, Timelsena K, Tamrakar M, Rosenberg R, Tuladhar S: **Equity Analysis of Maternal Health Services in Nepal: Trends and Determinants, 2011–2022 Nepal DHS Surveys**. In.; 2024.
23. Khanal GN: **Conditional cash transfer policies in maternal health service utilization in Nepal: Analysis of safe delivery incentive program (Aama Surakshya Karyakram) using Kingdon's multiple streams framework**. *Int J Health Plann Manage* 2019, **34**(1):e131-e141.
24. MOHP [Nepal]: **Maternal and newborn care incentive program guideline**. In. Edited by Division FH. Kathmandu, Nepal; 2020.
25. [Nepal] M: **Standard treatment protocol (STP) for basic health services (BHS) package 2078**. In. Edited by Division FH. Kathmandu, Nepal; 2020.
26. Devasenapathy N, Neogi SB, Soundararajan S, Ahmad D, Hazra A, Ahmad J, Mann N, Mavalankar D: **Association of antenatal care and place of delivery with newborn care practices: evidence from a cross-sectional survey in rural Uttar Pradesh, India**. *J Health Popul Nutr* 2017, **36**(1):30.
27. Marmot M: **Just societies, health equity, and dignified lives: the PAHO Equity Commission**. *The Lancet* 2018, **392**(10161):2247-2250.
28. World Health Organization: **A conceptual framework for action on the social determinants of health**. In. Geneva: World Health Organization; 2010.
29. Cao WR, Shakya P, Karmacharya B, Xu DR, Hao YT, Lai YS: **Equity of geographical access to public health facilities in Nepal**. *BMJ Glob Health* 2021, **6**(10):e006786.
30. Khatri RB, Karkee R, Durham J, Assefa Y: **Universal coverage of the first antenatal care visit but poor continuity of care across the maternal and newborn health continuum among Nepalese women: analysis of levels and correlates**. *Globalization and Health* 2021, **17**(1):1-15.
31. Sserwanja Q, Musaba MW, Mutisya LM, Olal E, Mukunya D: **Continuum of maternity care in Zambia: a national representative survey**. *BMC Pregnancy Childbirth* 2021, **21**(1):604.
32. Rahaman M, Roy A, Chouhan P, Malik NI, Bashir S, Ahmed F, Tang K: **Contextualizing the standard maternal continuum of care in Pakistan: an application of revised recommendation of the World Health Organization**. *Front Public Health* 2023, **11**:1261790.
33. Roder-DeWan S, Nimako K, Twum-Danso NAY, Amatya A, Langer A, Kruk M: **Health system redesign for maternal and newborn survival: rethinking care models to close the global equity gap**. *BMJ Glob Health* 2020, **5**(10):e002539.
34. Hanson C, Schellenberg J: **Redesigning maternal health services: is centralisation the answer in low-resource settings?** *BMJ global health* 2019, **4**(3):e001488.
35. McRae DN, Bergen N, Portela AG, Muhajarine N: **A systematic review and meta-analysis of the effectiveness of maternity waiting homes in low- and middle-income countries**. *Health Policy Plan* 2021, **36**(7):1215-1235.
36. Ministry of Health and Population: **ANC to PNC continuum of care guideline**. In. Kathmandu, Nepal: Ministry of Health and Population,; 2021.

37. Mothupi M, Dasgupta J, Hosseini Jebeli SS, Stevenson J, Berdichevsky K, Vong S, Barasa E, George A: **Using an intersectionality approach to transform health services for overlooked healthcare users and workers after covid-19**. *BMJ* 2023, **381**:e072243.
38. Khatri RB, Dangi TP, Gautam R, Shrestha KN, Homer CSE: **Barriers to utilization of childbirth services of a rural birthing center in Nepal: A qualitative study**. *PLoS One* 2017, **12**(5):e0177602.
39. Riese S, Dhakal R: **Understanding the Three Delays among Postpartum Women at Health Facilities in Nepal**. In*.* Kathmandu, Nepal; 2023.
40. Karkee R, Lee AH, Binns CW: **Bypassing birth centres for childbirth: an analysis of data from a community-based prospective cohort study in Nepal**. *Health policy and planning* 2015, **30**(1):1-7.
41. Karkee R, Kadariya J: **Choice of health-care facility after introduction of free essential health services in Nepal**. *WHO South East Asia J Public Health* 2013, **2**(2):96-100.
42. Khanal GN, Bharadwaj B, Upadhyay N, Bhattarai T, Dahal M, Khatri RB: **Evaluation of the National Health Insurance Program of Nepal: are political promises translated into actions?** *Health Research Policy and Systems* 2023, **21**(1):7.
43. Mahat A, Citrin D, Bista H: **NGOs, partnerships, and public-private discontent in Nepal's health care sector**. *Medicine Anthropology Theory* 2018, **5**(2).
44. Ensor T, Bhatt H, Tiwari S: **Incentivizing universal safe delivery in Nepal: 10 years of experience**. *Health Policy Plan* 2017, **32**(8):1185-1192.
45. Betran AP, Torloni MR, Zhang JJ, Gulmezoglu AM, Section WHOWGoC: **WHO Statement on Caesarean Section Rates**. *BJOG* 2016, **123**(5):667-670.
46. Laxmi T, Goma D, Kumariniraula H, Roshnitui T, Binod A: **Rising cesarean section rates in Nepal: question of safety and integrity on obstetric emergency practice**. *J Gynecol Women's Health* 2017, **7**(4):555716.
47. Singh DR, Jha T: **Exploring factors influencing antenatal care visit dropout at government health facilities of Dhanusha District, Nepal**. *American Journal of Public Health Research* 2016, **4**(5):170-175.
48. Kc A, Malqvist M, Bhandari A, Gurung R, Basnet O, Sunny AK: **Payment mechanism for institutional births in Nepal**. *Arch Public Health* 2021, **79**(1):163.

Click here to access/download;Figure;Figure 1.docx

Figure 1

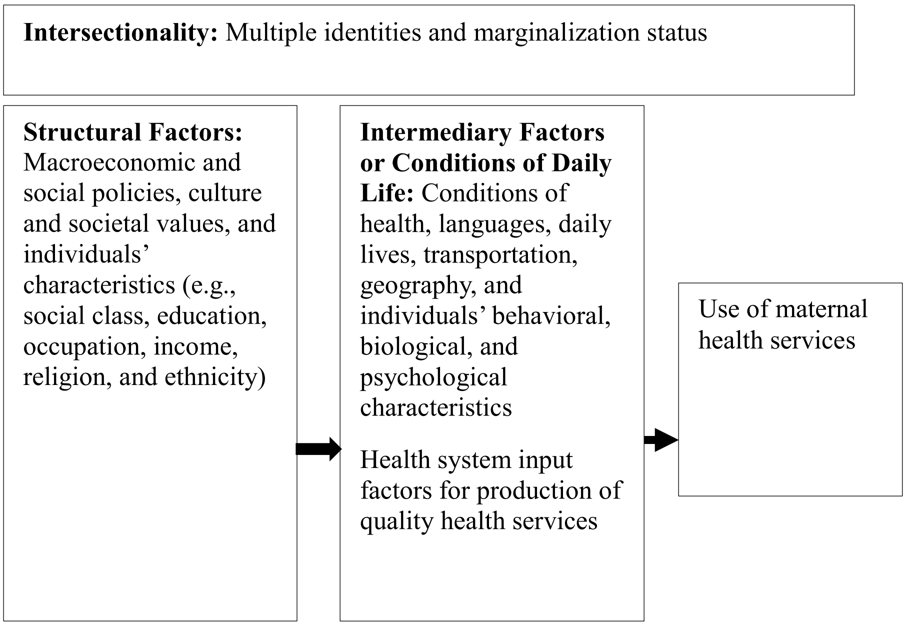

Figure 2

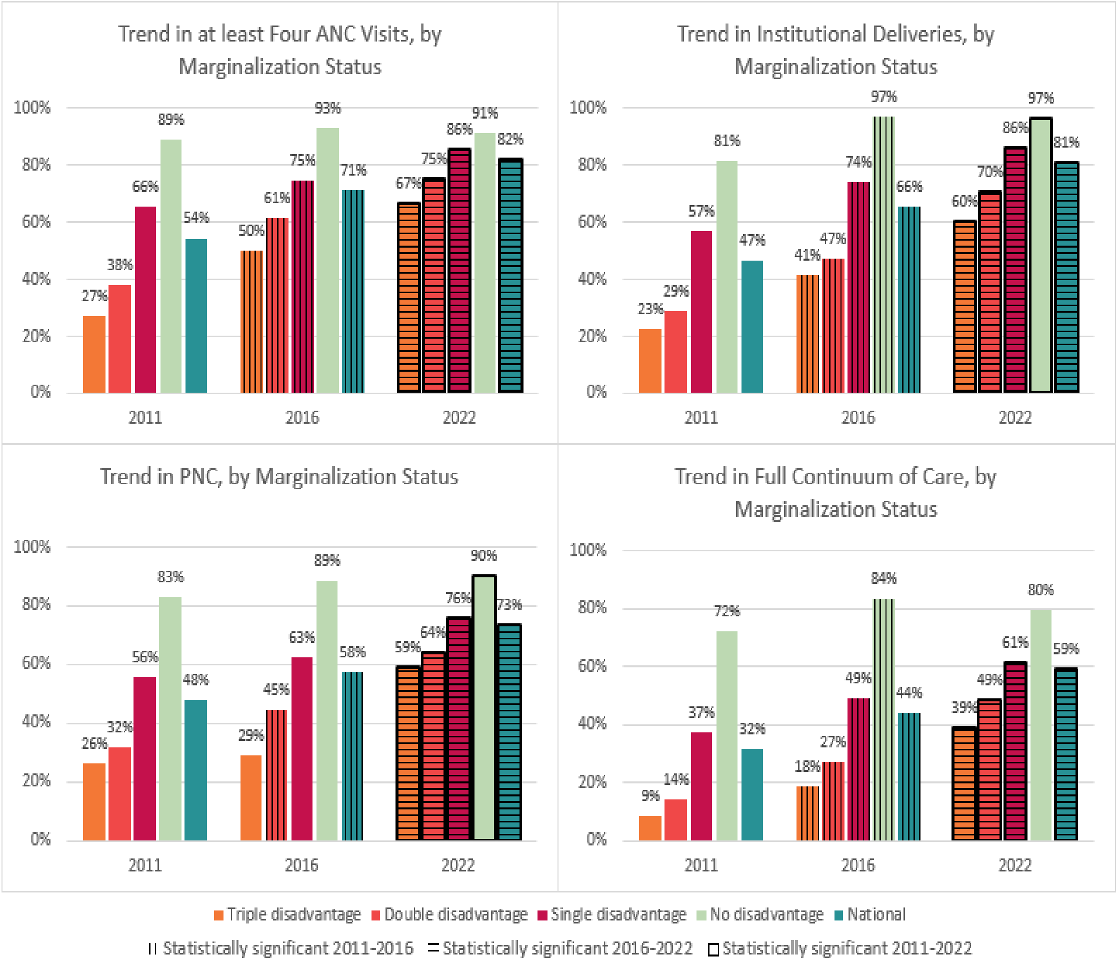
Trend in at least Four ANC Visits, by Marginalization Status
Trend in Institutional Deliveries, by Marginalization Status
Trend in PNC, by Marginalization Status
Trend in Full Continuum of Care, by Marginalization Status
0%
20%
40%
60%
80%
100%
2011
2016
2022
Triple disadvantage
Double disadvantage
Single disadvantage
No disadvantage
National
Statistically significant 2011-2016
Statistically significant 2016-2022
Statistically significant 2011-2022

Click here to access/download;Figure;Figure 3.docx

Figure 3

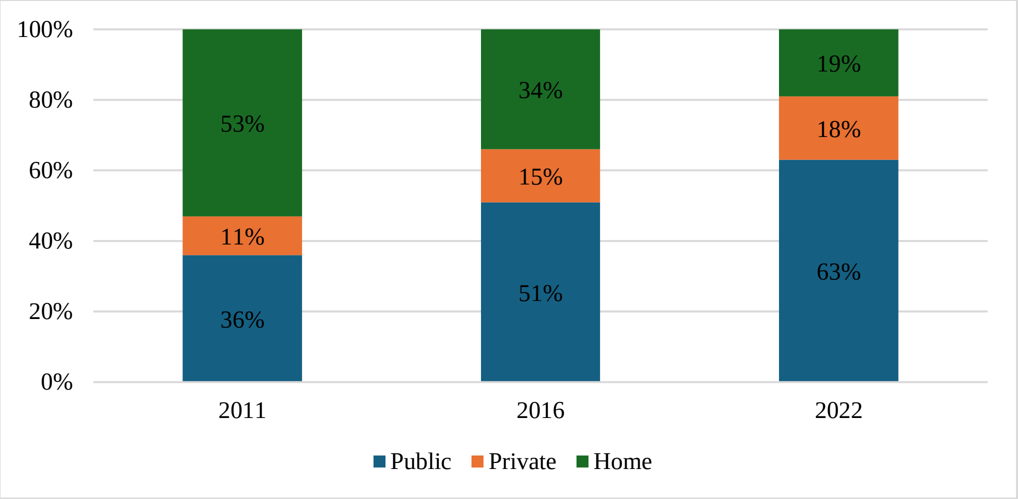

Figure 4

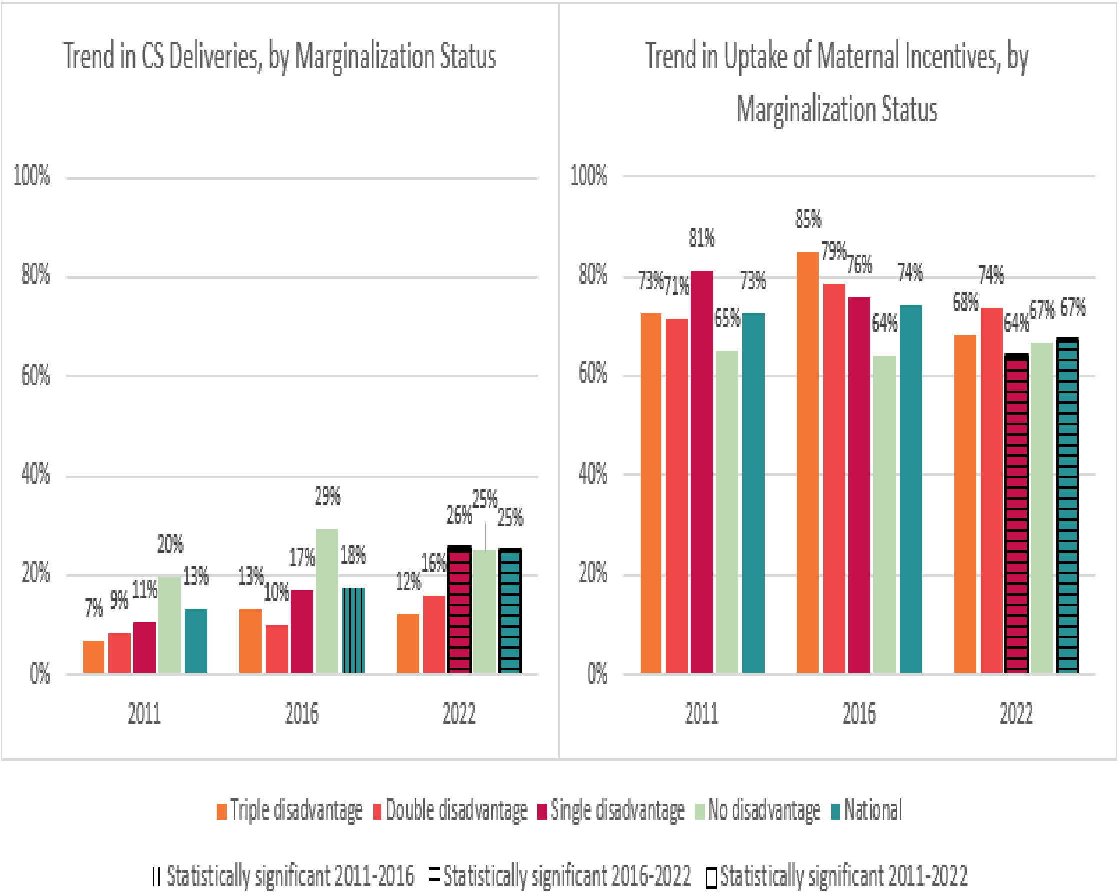
Trend in CS Deliveries, by Marginalization Status
100%
80%
60%
40%
20%
0%
7%
9%
11%
20%
13%
13%
10%
17%
29%
18%
12%
16%
26%
25%
25%
2011
2016
2022
Trend in Uptake of Maternal Incentives, by Marginalization Status
73%
71%
81%
65%
73%
85%
79%
76%
64%
74%
68%
74%
64%
67%
67%
Triple disadvantage
Double disadvantage
Single disadvantage
No disadvantage
National
Statistically significant 2011-2016
Statistically significant 2016-2022
Statistically significant 2011-2022

Click here to access/download;Figure;Figure 5.docx

Figure 5

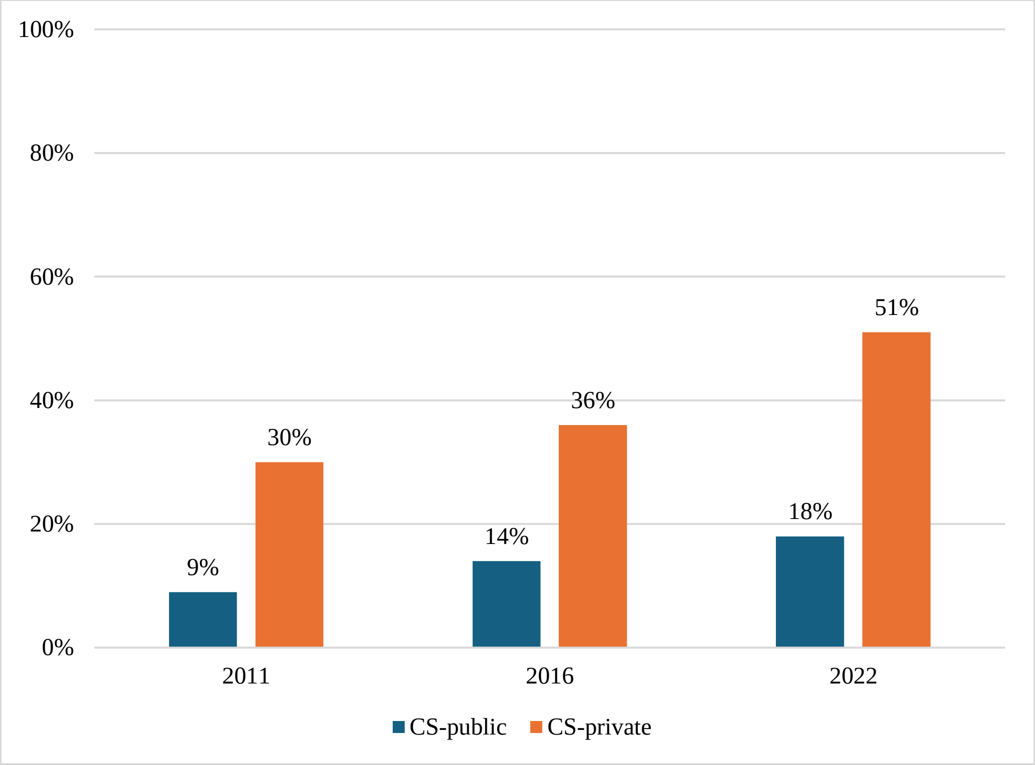

Click here to access/download;Figure;Figure 6.docx

Figure 6

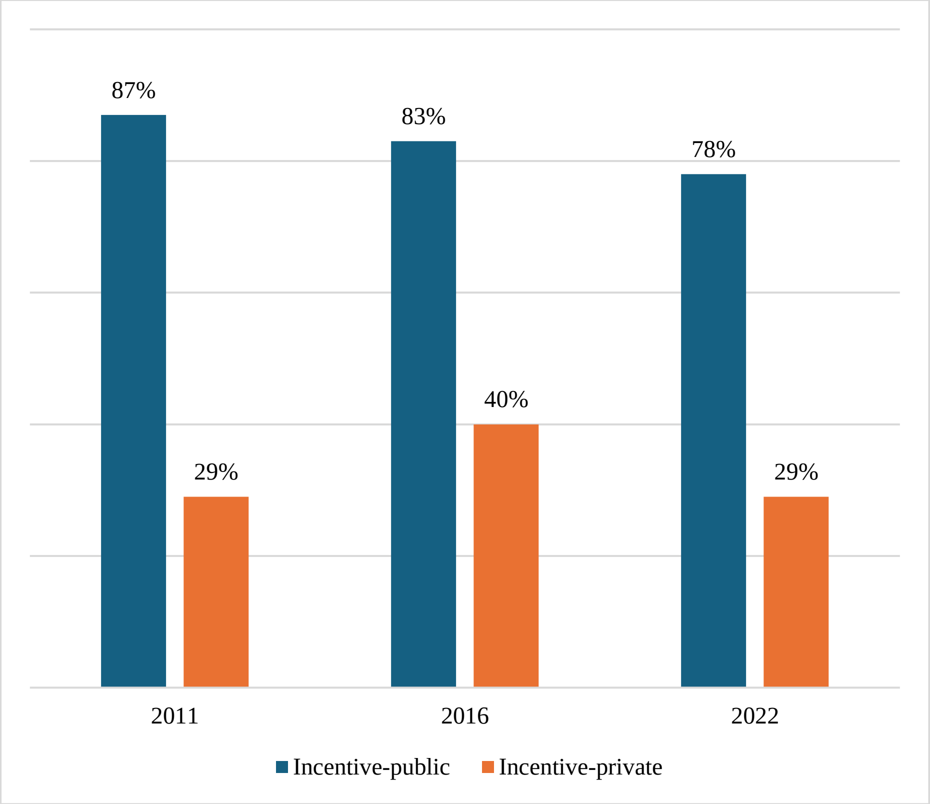

Supplementary information

Click here to access/download
Supporting Information
Supplimentary information MH.docx
