# Supplementary material for: Unpacking equity trends and gaps in Nepal’s progress on maternal health service utilization: Insights from the most recent Demographic and Health Surveys (2011, 2016 and 2022)
Source: PLoS One. 2025 Nov 20;20(11):e0337434. doi: 10.1371/journal.pone.0337434 (PMC12633875; doi:10.1371/journal.pone.0337434)
Supplement: Attachment — Submitted filename: MaternaHealth_Nepalstudy_Review_PS.docx [file pone.0337434.s003.docx]

Review suggestions:

1. Title of the slide needs a revision, maybe adding years and then rewording might give more clarity. I am not clear on this as “Determinants of institutional delivery” includes the year 2022 NDHS data set only.
2. Line 28 in abstract section: “Completion of all maternal needs” required to be specified as it sounds like it comprises of everything that author has already mentioned (at least four antenatal care (ANC) visits, institutional deliveries, first postnatal care (PNC) within 48 hours of childbirth).
3. Line 32-47, Results in abstract section:
   a. The paragraph is dense and strings multiple findings together. Breaking into sub-paragraphs by theme might help readers to understand the results summary clearly.
   b. Findings are described qualitatively (“increased odds”, “reduced rates”) without any quantitative estimates, making it difficult to understand by how much.
   c. Missing total subjects included in the analysis, as 2 different aims have different sample size. You have mentioned in the methods section, but it is not clear if all the samples are included or not in the final analysis
   d. Line 40: completion rates used here is a bit vague, this may require specific definition in an appropriate space
   e. Line 43: “women engaged in manual labor” means two different things to me, please reword this phrase to make it more clear on what you are trying to say
   f. Instead of increased odds or reduced odds, consider using higher or lower
   g. Some sentences are very long (esp. lines about multivariable analysis). Breaking them into shorter, sharper statements will improve readability.
   h. What are “disadvantaged ethnicities” in line 38?
4. Conclusion section (line 48-57)
   a. Sentences are long-rephrase for clarity.
   b. Recommendations (Lines 54–57) should be specific and findings-driven.
   c. Reword “nonexistent maternal incentives” to something clearer.
5. Keywords (59-60):
   a. Please arrange the keywords in alphabetical order as per journal conventions.
   b. Additionally, consider prioritizing terms based on their frequency and centrality in your study
   c. Ensure consistency with terminology used in the abstract
6. Introduction:
   a. Line 69-70 says increased but does not explicitly mention what kind of increase (quantitative information is missing)
   b. Line 73 to 78 and check throughout if missed in remaining sections too: recheck the referenced paper and mention the unit for MMR and NMR numbers
   c. Line 74: Citation is out of order, consider reordering. And recheck the reference and the referred article for citation number 21 here, as content does not match with the cited paper
   d. Revise all the citation numbers, also check particular contents from appropriate references
   e. Once abbreviated, it is redundant to abbreviate in the following texts, revise accordingly
   f. Line 83 to 85 mentions the percentages and it would be nice to learn out of how much (denominator)
   g. Line 88, not so sure if 10^th^ citation really reflects the information explained in that particular sentence. Check if referencing and cited sentences are related or not
   h. Line 89: could not understand the meaning of contact coverage? Is there any better way to explain it?
   i. Line 119, 124: Aama program (maternity incentive program), check the specific name for this program as this is widely popular program too
   j. Line 120: Transportation costs meaning? Payment, reimbursement, partial payment? Any specific amount?
   k. Authors gave detailed stats (MMR, NMR, inequalities, Aama details, federalism, private sector shifts) before that please state clearly what’s missing in research. Hence, making readers not able to grab the study gap. Revise sequencing the sentences
   l. Equity gaps by wealth, education, ethnicity, and provinces are repeated in different places.
   m. Ensure terms like *“maternal incentive”* vs. *“maternity incentive program”* are consistent.
7. Aims of the study: The aims of the study have been expressed differently in the abstract section and introduction section, as well as need to synchronize with the title. Please revise accordingly
8. Methods:
   a. Line: 145-146: cite the link of DHS consistently, this can be listed in the reference list too. If there is specific link to the particular dataset, use those links
   b. Elaborate explanations on study design, settings, study participants (eligibility criteria), sample size including sampling technique. Flowchart is helpful to understand how the participants are included in the study, or detail explanation is required. If different aims have different study settings, explain them clearly too
   c. Line 149-150: Please justify why only one year prior to each selected NDHS was selected.
   d. Mention in separate section or elaborate about the potential confounders.
   e. Line 151-152: For 796 (weighted), if weighted meaning weighted, this should be explained in findings than methods section. As weighed analysis is done only after analysis.
   f. Study variables: explain dependent and independent variables in separate headings for more clarity. While explaining the variables, refer to the particular aims for better understanding to the readers
   g. Please explain well on how the outcome/dependent variables were measured.
   h. Provide rationale for dichotomization of key variables and consider sensitivity checks.
   i. Line 161 and 162: For the definition of marginalization status, why it has been adapted from the study in India? Mention a line to justify this
   j. Line 162-168, clearly explain which steps are those.
   k. The term disadvantage has been used several times, and other terminologies also include this disadvantage terminology, which creates confusion while reading this. This needs to be presented more clearly
   l. How is wealth quintile measured? Mention this in detail, since this is also one of the key variables used in the study. You may explain this is S1 table if there is space limitation
   m. Please clearly mention the definition of the four categories of disadvantage status: triple, double, single, and no disadvantages
9. Conceptual framework: While the framework is presented, it is not entirely clear how it was applied to variable selection or analysis. Could not follow how this conceptual framework was applied and coordinated with other sections of the study.
10. Data analysis (line 188-198):
    a. The last sentence sounds like author used STATA only for the survey, so make it clear by rewording the sentences
    b. The analysis methods are expressed in general structure, please specify which tests were used for which purposes. How many models are used? Which analysis were conducted for aim 1 and which ones for aim 2, explain in detail. Please specify statistical tests specifying the use of it in specific variables.
    c. For adjusted analysis, please explain which covariates were adjusted
    d. DHS datasets usually have some missingness. Did you exclude missing cases listwise, or apply imputation? This should be mentioned for transparency.
    e. Since the study emphasizes intersectionality (triple/double disadvantage), reviewers may expect clarification on whether interaction terms were tested in regression models, or whether disadvantage status itself captures these interactions.
11. Research ethics:
    a. Line 203-204: Does this study need to mention about the informed consent by mothers on behalf of their children? The study participants sentence does not explain it includes children, or it does, explain this clearly in the study participants’ section
12. Results section:
    a. Any particular reason to compare national level trend in the findings, if not, discussion section is a better space to do all these comparisons. In the finding section, the study’s findings should get highlighted. Check this for following sections throughout findings section
    b. Line 218-219: Requires quantitative information as well.
    c. Line 221-224: what does those statistically significant statements reflects, if authors have used any statistical tests, provide some numbers as well as significant values. Need clarity on what statistical test determined “significance.”
    d. Line 209-227: the findings are more subjective, please explain it in a quantitative way
    e. Lin 223: after NDHS looks incomplete. NDHS???
    f. Line 242: cs should be capitalized
    g. Line 261-268: Could not compare with the presented graph in figure 6.
    h. Line 271-276: What is the total sample size for those percentages used?
    i. Table 1: Which test gave CI? Mention about this under the table as a “Note”
    j. Retotal % in table one as it required to total to 100%, wherever appropriate
    k. Line 313 – 333, mention variables those were adjusted. Authors may use the space below table as a footnote. Follow this for the following paragraphs too
13. Discussion
    a. Add strength of the study portion
    b. Reorder the discussion section as follows:
       i. Main findings (summary in 2–3 paragraphs).
       ii. Comparison with existing literature (situating Nepal in regional/global context).
       iii. Strengths & limitations
       iv. Implications for policy & practice
    c. In the limitations: the sentences are *dense and technical*. I prefer clear, plain-language explanation of how these limitations might affect results of the study
    d. Report other relevant limitations (cross sectional design, self-reported data, no quality-of-care indicator, language and cultural barriers) explaining how it distorts study findings.

Figures:

a. Figure title: Clear label missing
b. For graphs: Label axes to reduce clutter (avoid repeating % signs).
c. Figure 2: colors used are less distinct, consider making it more visible
d. Label the axis for more visual expressions, this will avoid use of several % signs in 1 figure
e. Figure 4: add title, labels and omit % in the graph.
