# Supplementary material for: Unpacking equity trends and gaps in Nepal’s progress on maternal health service utilization: Insights from the most recent Demographic and Health Surveys (2011, 2016 and 2022)
Source: PLoS One. 2025 Nov 20;20(11):e0337434. doi: 10.1371/journal.pone.0337434 (PMC12633875; doi:10.1371/journal.pone.0337434)
Supplement: Attachment — Submitted filename: Recommendation_Final.docx [file pone.0337434.s004.docx]

**Limitation and Recommendations on the study titled, " Trends and Determinants of Maternal Health Service Utilization in Nepal: Insights from the Most Recent Three Demographic and Health Surveys".**

**Limitation**

- **Missing Figures and Tables**: This makes it hard to verify / understand the results, like trends by marginalization status and conceptual framework.***[Results sections].***

**Recommendations:**

- **It would show more meaningful results if switched to Relative Risks (RRs) for Common Outcomes**: Logistic regression odds ratios are reported, but some outcomes are quite common (>50%), so odds ratios may exaggerate effect sizes. Relative risks could have been more appropriate for those outcomes. ***[Methods, Results sections].***
- **Disadvantaged Categories:** The study creates a "disadvantage status" variable by combining education, wealth, and ethnicity into four groups (triple, double, single, or no disadvantages). To do this, it simplifies these factors into two categories each, like splitting wealth into lower 40% (poorest) and upper 60% (richer). This simplification makes the data easier to analyze but loses important details and it treats all poor people the same, even if some are much poorer than others. Because the new variable is made from these three factors, they overlap too much (collinearity), so the study couldn’t include education, wealth, or ethnicity separately in the analysis. This hides how each factor affects things like hospital deliveries on its own, making it harder to pinpoint specific problems or solutions. However, it is included in Limitation section. ***[Methods section].***
- **Expand the Limitations Section**: Add details on NDHS biases (e.g., "Self-reported data may cause recall bias for ANC/PNC timing") and incentive analysis issues (e.g., "Including facilities where Aama Program was not available may underestimate uptake"). This change can make the study more transparent ***[Limitations section].***
- **Policy Recommendations**: Recommendations are broad (e.g., Regulate private sector, expand Aama program). The recommendations would benefit if it is more sharper, sepcific and actionable guidance***. [Recommendation Section]***
