# Supplementary material for: Unpacking equity trends and gaps in Nepal’s progress on maternal health service utilization: Insights from the most recent Demographic and Health Surveys (2011, 2016 and 2022)
Source: PLoS One. 2025 Nov 20;20(11):e0337434. doi: 10.1371/journal.pone.0337434 (PMC12633875; doi:10.1371/journal.pone.0337434)
Supplement: Attachment — Submitted filename: review_maternal health.docx [file pone.0337434.s005.docx]

The authors have well prepared the manuscript. There are few questions and suggestions from my side.

Line 23: I think the word ‘analyses’ should be replaced with ‘analysis’.

Line 27: Please explain how did you assess the PNC visit within 48 hours. Government has protocol of 24 hours, 3^rd^ day and 7^th^ day.

Results section in abstract: The interpretation of the major findings would be clearer with the numerical value. It is difficult to understand how higher or lower is the problem mentioned. State the percentage or odds ratio where required. Is caesarean section a major variable in the study and a part of maternal continuum?

Line 46: Is there provision of providing maternity incentive by the private hospitals?

236-237: Clarify whether the home delivery is 18 or 19% in 2022.

242: Write cs in capital letter

Can you provide reference for the recategorization of independent variables such as ‘marginalized status and disadvantages’ if any.

244: Rearrange a sentence as ‘at least a live birth in a health facility’.

278: Table 1, it would be better to present the distribution of weighted women rather than unweighted or You can present the frequency of both. In the same table, is ‘age in year’ the current age of women or the age of women at childbirth? Please state clearly. If it is the current age, should be mentioned at first component of the demographic characteristics.

The findings show the doubling of CS in public facilities too, which you haven’t emphasize. Since the size of institutional delivery is too large in public facilities, the percentage might have seen less here. Whereas the delivery in private facilities is less compared to public so that CS seems higher there. Please clearly describe it.

The interpretation and discussion are okay. Conclusion and recommendations are well written.

Ensure all the components according to STROBE checklist.
