# Supplementary material for: Unpacking equity trends and gaps in Nepal’s progress on maternal health service utilization: Insights from the most recent Demographic and Health Surveys (2011, 2016 and 2022)
Source: PLoS One. 2025 Nov 20;20(11):e0337434. doi: 10.1371/journal.pone.0337434 (PMC12633875; doi:10.1371/journal.pone.0337434)
Supplement: Attachment — Submitted filename: Review MH Service Utilization.docx [file pone.0337434.s006.docx]

**1. Inconsistent Sample Sizes**

The manuscript's "Methods" and "Results" sections present conflicting information regarding the sample size used for the determinant analysis. The abstract and "Methods" section state that the determinant analysis for institutional delivery was performed on the 2022 NDHS dataset. However, the "Methods" section specifies that the total sample size for the determinant analysis of institutional delivery, delivery by CS, and maternal incentives was 796 women who gave birth at a health facility. In contrast, the "Results" section states that the descriptive analysis of institutional delivery was based on 981 study participants. This discrepancy in sample sizes between the methodology and the results section for the same analysis is unclear.

**2. Unclear Explanation of the "Disadvantage Status" Variable**

The manuscript explains that a new variable, "disadvantage status," was created by combining three background variables: education, wealth status, and ethnicity. It details the process of dichotomizing these variables and then merging the categories. The final variable is said to have four categories: triple, double, single, and no disadvantages. However, the description of how the categories with "at least one form of disadvantage" and "two forms of disadvantage" were merged is confusing and does not clearly explain which specific combinations of the original eight categories were merged to create the final four. This lack of clarity makes it difficult to replicate the analysis.
