# Supplementary material for: Unpacking equity trends and gaps in Nepal’s progress on maternal health service utilization: Insights from the most recent Demographic and Health Surveys (2011, 2016 and 2022)
Source: PLoS One. 2025 Nov 20;20(11):e0337434. doi: 10.1371/journal.pone.0337434 (PMC12633875; doi:10.1371/journal.pone.0337434)
Supplement: Attachment — Submitted filename: Paper Review - PLOS One .docx [file pone.0337434.s007.docx]

**Overall Impression of the Paper:**

The paper looks well structured and has provided a glimpse of health inequity and its effects in one of the most important areas, maternal service utilization.

**Strengths of the Paper:**

- Wise use of NDHS data that represent the whole nation
- Has provided a good rationale and aim

**Areas to improve:**

**Abstract:**

The abstract looks quite lengthy, consider it concise.

**Methods:**

- In the Methods section (line number 151-153) under data sources, the sample size and weighting could be explained quite a little more to make the readers unfamiliar with this concept clearer.
- In the study variables under methods, section, explanation of outcomes and exposures could be done in separate paragraphs under these headings to make the picture clear.
- Provide reference for advantaged ethnicities.
- Creation of marginalization status and disadvantaged status could be better explained using a flowchart.
- Figures are yet to be inserted, so it makes the concept cloudy at places.

**Results:**

The results are well presented but contain many tables, consider merging the tables where relevant. Also highlight the major findings in tables so that the navigation gets easier. If possible, show 2-3 results in a single graph using multiple colors to indicate different findings’ trend and provide indices.

**Discussion:**

- Line number 404 and 405: Terminologies such as catch up rate and keep up rate need to be defined. Also, define what are intersectional groups.
- Discussion is done in different paragraphs for different findings which makes things clear and reading easier but considering the length of all the paragraphs can be done to make it concise.
- Policy implications are written in a separate paragraph, well-done. Adding recommendations on indications for CS procedure monitoring specially in private health facilities and also providing incentives under Aama Program even in private HFs with reference can be valuable.
- In the limitations section, adding the caution for interpretation on a small subgroup (Muslim Dalit, Mountainous regions) can be done.

**Conclusion** is well-written, consider it breaking down to two paragraphs: Conclusion and Recommendations
