# Supplementary material for: Unpacking equity trends and gaps in Nepal’s progress on maternal health service utilization: Insights from the most recent Demographic and Health Surveys (2011, 2016 and 2022)
Source: PLoS One. 2025 Nov 20;20(11):e0337434. doi: 10.1371/journal.pone.0337434 (PMC12633875; doi:10.1371/journal.pone.0337434)
Supplement: Attachment — Submitted filename: Response to reviwers_R1.docx [file pone.0337434.s008.docx]

## Comments from the editor

Comment: Please work on abstract section to highlight the importance of the paper for global readers, low- and middle-income countries and why such study is important.
Response: some global and regional context is added to draw the attention of global readers.

Comment: In background section of the manuscript, some information should be added. The paper only highlights national importance. I suggest adding more information about global, regional and especially LMICS country context.
Response: Thanks for the global and regional context of maternal health has added in the introduction section.

Comment: In method section, how did you work with sample weight for three rounds of the survey? Please indicate it.
Response: Weighted sample was identified using the sampling weight given in the individual survey for specific year. There is no need to adjust the sampling weight of pooled data of three surveys. This has been indicated in the revised manuscript. Please look for detailed analysis code in [DHS-Analysis-Code/Analysis_Reports/FA152_maternalhealthequity/FA152_MaternalHealth_71824.do at main · DHSProgram/DHS-Analysis-Code · GitHub](https://github.com/DHSProgram/DHS-Analysis-Code/blob/main/Analysis_Reports/FA152_maternalhealthequity/FA152_MaternalHealth_71824.do)

Comment: Line 196- You mention something about Stata command. How does it address all the statistical calculations for 3 rounds of the survey?

Response: The descriptive analysis of individual survey was calculated and presented the trends of each outcome variable. Their significance was tested using z-test for proportion taking data from individual survey using two sample significance difference of proportion (proportion test). While multivariable logistic analysis was conducted for the data of the NDHS 2022 only. Please look for code used in this analysis ; [DHS-Analysis-Code/Analysis_Reports/FA152_maternalhealthequity/FA152_MaternalHealth_71824.do at main · DHSProgram/DHS-Analysis-Code · GitHub](https://github.com/DHSProgram/DHS-Analysis-Code/blob/main/Analysis_Reports/FA152_maternalhealthequity/FA152_MaternalHealth_71824.do)

Comment: Did you use any checklist such as STROBE for the present analysis? If yes, where is it? If not, why? Please include information for all three rounds of study.

Response: This study was based on the further analysis of publicly available secondary data. Yes, we followed most of the steps of STROBE checklist to report the findings and draft this manuscript.

Comment: Total sample= Year I, Year II, Year III, excluded= Year I, Year II, Year III,
Included =Sample= weighted vs non-weighted in analysis?

Response: As mentioned above, we used the secondary data of NDHSs, we briefly mentioned that how NDHS designed sampling procedure and how final respondents are selected for the interview, and how information is collected. Detailed sampling size and methods is described in the original reports of each NDHS, we have referenced them for further readings for interested readers. More specifically, NDHS collected information related to maternal health care utilization among mothers who had live birth five years prior to the survey. But in our analysis, we included women who had live birth one year prior to each survey. We have mentioned that all sample included in the analysis are weighted otherwise specifically indicated.

Comment: Ethics Section-Please include how ethical approval was obtained. In the latest survey, there was COVID-19 how it was addressed. Please cite the relevant DHS report indicating ethics information.

Response: this is further analysis of secondary data derived from the nationally representative surveys in Nepal – i.e. Nepal Demographic and Health Surveys (2011, 2016, and 2022). Ethical approval for those surveys was described in their original reports which we have cited in the methods section of our manuscript. In our manuscript, we have briefly described on how ethical procedure occurred in the NDHS survey. For this analysis, the researcher team was granted approval from ICF to download and use data for the further analysis.

Comment: Results-Table 1. provides only information about 2022 survey, is it correct? And how about N or n here?
Response: table 1 provides the distribution of sample included in the study. Descriptive analysis of the sample is always calculated as column percentage. In our analysis, for NDHS 2022, N was 981 for all variables, and numbers of sample for each variable categories (n) are reported in each row of each variable. In the revised table, N is included in each study variable as well.

Comment: Table 2-5, you mention about AOR for different determinants. Among these, which are the most powerful factors? And how have the impacts of these factors changed over time? For example, the impact of education, family size, husband occupations were most important factors in 2012 (let’s say model I). How does it change over the time in 2022. Are the factors the same?

Response: the analysis output included in tables 2-5 represents the outputs of binomial logistic regression analysis. Our study objective was to analyse the trends of maternal health service utilization over three surveys at the national level and by marginalization status. We examined the determinants of maternal health service analysis using data of NDHS 2022 only. We did not investigate the trends of change in associated determinants over time or most powerful determinants impacting the maternal service utilization as this was not our study objective.

Comment: Discussion-You stated about the significant inequalities among the groups. Is it based on all the data set of DHS or only based on 2022 information.

Response: Our study objective was to identify the associated determinants of maternal health service utilization using data of NDHS 2022.

Comment: Major comments: Your paper profoundly stated about equity gap, but nowhere in method section you stated about any test of equity. Please write about the equity test you performed and indicate relevant figure based on the equity test such as Lorenz Curve or others?

Response: we investigated the uptake of the maternal service utilization among women with multiple forms of marginalization (composite intersectional variable created using wealth status, ethnicity and education status- see our published paper based on NDHS2016, https://doi.org/10.1186/s12889-021-11142-8). In the current analysis, detailed of this approach is described in methods section and reported finding. Our equity analysis approach was different than conventional equity analysis (e.g. Lorenz curve or concentration index).

Comment: Data analysis section: You indicate VIF less than or equal to 5, what is the basis for such inclusion. You can present the VIF test results as supplementary file which will guide further researchers on how to look at VIF results.

Response: All Stata codes including calculation of VIF used to analyse this paper are publicly available in the GitHub webpage where researchers can access there. Here is the link of GitHub: [DHS-Analysis-Code/Analysis_Reports/FA152_maternalhealthequity/FA152_MaternalHealth_71824.do at main · DHSProgram/DHS-Analysis-Code · GitHub](https://github.com/DHSProgram/DHS-Analysis-Code/blob/main/Analysis_Reports/FA152_maternalhealthequity/FA152_MaternalHealth_71824.do)

Comment: Did you perform any factorial analysis? What is the basis of performing or not performing such analysis? You have included a lot of information for analysis, among them which are the most important factors, how has its role changed over time?


Response: As described in response to one of the comments above, our study objective did not require conducting factorial analysis. So, we did not conduct factorial analysis. Our study objective to look the descriptive trends of maternal service utilization in the last three survey (overall, and by marginalization status), and associated determinants of maternal health service utilization in NDHS 2022. There are several studies have already available which examined the determinants of maternal health services utilization data of previous NDHSs i.e. NDHSS 2011, or 2016.

Comment: Figure 1. does it represent your study flow? What is the theoretical basis for such a conceptual framework creation such as any health belief model or any models? Why do people come to utilize health services?
Response: Figure 1, a conceptual framework (which is different than analytical framework) is a thinking framework to guide our analysis. Our study is further analysis of secondary data and identify trends of maternal health services utilization among women in Nepal. As social determinants of health framework conceptualize, the underpinning idea behind health equity are founded on the structural, intermediary, and health system factors. Health equity depends on the structural inequity (such as education, ethnicities or wealth status) which are generally non-modifiable by health systems or non-health sector interventions (developmental approaches). Thus, to address the structural factors towards health equity, political and structural reforms are needed such as addressing poverty, ethnic inequalities. Health systems can provide health service to poor but cannot reduce poverty or improve wealth status. Intermediary factors are those factors which can be modified by development approaches (such as improving living, working, and travel conditions by making bridges or road etc). In our analysis, we consider geography or province as intermediary factors, if we improve road networks or bridge in the river, then the distance to health facility can be reduced that improve the access health services. Health system factors that can address through health systems including service readiness, quality of care etc (Detailed in [Towards equity of maternal and newborn health services in Nepal - UQ eSpace](https://espace.library.uq.edu.au/view/UQ:c718705)). The figure 1, i.e., conceptual framework represents those underpinning ideas of social determinants of health frameworks, and previous publications including the publication of first author of this paper. We used conceptual framework to guide our analysis based on the available variables and data in the NDHS 2022 dataset.

Comment: Figure 2. is congested, you can mention about % in y axis and remove % in each data text inside figure.
Response: figures have been revised and edited as suggested.

Comment: You can rework to make clear the data reported in each figure. Was the figure based on weighted average or not? Do you want to show CI for each piece of information?

Response: All data were weighted otherwise indicated. Figures will become more congested if we include the CI. So, we have kept data level as it is.

Comment: Uniformity in figure design is missing. Please include y- axis as percentage and make the figure less congested.
Response: figures have been revised and edited as suggested.

Comment: Figure 6. Your y-axis title is missing.

Response: figure have been revised and edited as suggested.

## Reviewer #1:

Comment: This paper presents a strong, equity-focused analysis of maternal health service utilization trends in Nepal using three NDHS datasets, with an innovative intersectional disadvantage index that adds policy relevance. To further strengthen the manuscript, the authors should deepen theoretical framing (e.g., Three Delays Model), clarify data limitations, enhance discussion with more analytical depth and global comparisons, report statistical significance of trends, and expand on actionable policy implications tailored to Nepal’s federal health system.

Please see the attached paper for my suggestions.

Response: we thank reviewer for these insightful and border feedback. As this study used health equity framework as conceptual framework and framed the research to answer who are benefited from the service utilization and who are left behind. For this we used adapted and modified version of social determinants of health and frame paper accordingly. The model like Three Delays Model is also important to describe when and where maternal deaths occur, and such framework can be useful to describe the more grounded research using primary data, more specifically qualitative data. Our research question was to analysis the secondary data and answer the question such as who are seeking maternal health service and national level and group level. For which we took secondary data, and we conducted further analysis. If our research question was like why women are not seeking care, what factors are contributing at household or community level, why women are not reaching health facilities, or what factors are influencing while seeking care in hospitals, then models like Three Delays Model could be more appropriate model. However, in our analysis, the second component of Three Delays Model (Delays in reaching health facilities or health workers for maternal care) was interlinked in our analysis. We have added some statement why women are seeking care (due to geographical factors or due to multiple marginalization status), we have incorporated those arguments in our interpretation and discussion.

Comment: Abstract - The result lacks quantitative depth. Consider stating the percentage point increase in institutional delivery or CS. Clarify whether the decrease in maternity incentive use is statistically significant.

Response: Qualitative information was supported by quantitative information in the abstract. Only significant information was included in the abstract.

Comment: Introduction -No global comparison of trends in institutional delivery, CS, or incentive programs (e.g., how does Nepal compare to other LMICs?)

Response: We have included brief global and regional context of maternal health in the beginning of the introduction section.

Comment: Results -No statistical test results are reported for trend changes. Provide p-values for trend comparisons across years.

Response: output of the significance test results was reported in the figure. We have included the supplementary files and detailed results output including p values.

Comment: Findings on CS trends are important, but causes (e.g., medical indication vs. elective) are not discussed—this may lead to misinterpretation of high CS rates. So better to add the line like- “However, the NDHS dataset does not capture the medical indication for caesarean delivery (e.g., elective vs. emergency), limiting the interpretation of whether these increased CS rates reflect genuine obstetric need or are driven by other factors such as provider preference, financial incentives, or patient demand.” Please discuss this point in the discussion section as well.

Response: Thanks for the suggestions. The suggested statements have included and discussed in the discussion section. We have incorporated the causes of the CS. As per WHO estimates, only 5-15% of total deliveries require CS as our findings exceeds this estimates that means women in Nepal opt elective surgeries, but reasons could be different why they opt for that. This is one of the research questions for further exploration which have discussed in our paper (study implication section). There are data limitations such as the NDHS data collected information related to incentive where few private HFs implement Aama program, and there is no cross verification of CS whether that was elective or medically indicated for the CS. In the future, NDHS could include such questions to reduce the data limitations.

Comment: Discussion -This reads like a repetition of results. The first paragraph should instead summarize the meaning of findings, not just restate them.

Response: we revised the discussion section, and remove the repetitive content with the result section, and interpreted findings. Our framing of the discussion section, first paragraph with summary of key findings. As we have multiple outcome variables under the trends analysis and determinants of four outcome variables related to maternal health service utilization. In the subsequent paragraphs we interpreted findings on each key outcome variables and structured each paragraph taking key variables under study and compared and contract study findings with studies from other similar settings/ countries. We have included some subsections on strengths and limitations of the study, and implications of the study findings.

Comment: You should strengthen your discussion by connecting your findings to an established theoretical framework—in this case, the Three Delays Model—to better explain why women drop out of the maternal care continuum (e.g., from ANC to institutional delivery to PNC).

Response: We adopted the social determinants of health framework (structural, intermediary and health system factors towards equity) to frame our paper. The second component of three delay model (delays in seeking care) was interlinked in the discussion of maternal health care discontinuation from ANC to PNC visit.

Comment: The language barrier point is excellent. Based on this, you can add a recommendation: recruiting local auxiliary nurse midwives from under-served ethnic communities or language groups. Are there any similar kinds of programs implemented in other countries like India and Bangladesh to contextualize Nepal's Aama program?

Response: Thank you. We have added suggested recommendations and included similar evidence on another context if they can contexutalise in Nepali context for the Aama program. There are programs implemented to improve maternal health in Bangladesh (Maternal Health Voucher Scheme, Maternity Allowance Program) and India (Pradhan Mantri Matru Vandana Yojana) have been disused.

Comment: Also, it is better to discuss maternal health risks of unnecessary CS.

Response: thanks, we added on unnecessary use of CS or elective CS for the routine maternity care.

Comment: Group recommendations can be made for clarity:1. Structural and systemic reforms (e.g., roads, local hiring, cultural competence); 2. Program-level reforms (e.g., revised incentives, mandatory PNC, quality audits); 3. Regulatory/monitoring reforms (e.g., CS audits, private sector regulation, linking incentives to equity metrics).

Response: we have added suggested recommendations in the revised manuscript. These are important suggestions and useful

## Reviewer #2:

 Comment: Thank you for the opportunity to review this manuscript. The topic is very important and highly relevant currently. However, there are several areas for improvement:
Overall: The authors should avoid common patterns found in AI-generated text and consider redrafting the manuscript in more natural, human language. There are instances of long dash lines (-) appearing in many places, as well as an overuse of phrases such as "underscores" and "highlights."
Response: We have edited and proofread the revised manuscript multiple times and reduced the repeated phrases.

Comment: Here are some of my suggestions. Abstract: 1. years is missing in the sentence ".... among 25 women aged 15–49 who had experienced at least one live birth prior to each survey"; 2. Please include some key numbers in the results section.
Response: Survey year is mentioned earlier and “at least one year prior to survey” is included in the sentence. This means mother who had live birth prior to survey 2011 indicates those gave birth prior to NDHS 2011. Similar windows occur for other survey too.

Introduction
Comment: The introduction section appears to be too lengthy; it would be better to shorten it.
Response: we tried to write succinct and concise introduction section focussing on key context, literature gaps, and research objective. There are conflicting suggestions from other reviewers some suggest adding more. However, we agree with the reviewer that we have made succinct and concise introduction covering essential components of introduction section (context of research topic, and available evidence, research gaps, study objective and study significance).

Comment: You have talked about socioeconomic status here. Please also talk about the availability, accessibility, and equity gaps in maternal health services in Nepal.
Response: we thank reviewer for this feedback. We have stated some statement on availability, accessibility and equity of maternal health services.

Comment: There are several terms such as "marginalization," "disadvantage," and "multiple marginalization." Please reconsider these terms and ensure consistency throughout.

Response: we have made it consistent terms using as “marginalization status”.

Methods:
Comment: Please include details about the sample size, such as how DHS sampling was conducted. Additionally, specify the type of multivariate analysis performed, such as logistic regression. The tables also need footnotes to clarify the variables adjusted for in the multivariate models.

Response: We briefly explained the sampling methods used in DHS survey. The detailed sampling approach, data collection process are described in the original NDHS reports which we have cited in this manuscript. Given the large dataset of NDHS survey, we included women who had live birth one year prior the survey and indicated the sample size included in our analysis. Our focus in the methods section was what we did the selected sample data from NDHS rather than what NDHS has done (interested reader can go and look the NDHS reports).

We used multivariable regression analysis. In the regression analysis, we adjusted all variables included in the analysis. The variable included variable in the final model for each outcome are described in the data analysis subsection of methods section.

Results:
Comment: There are other categories in many tables, better to add what are included in that category.

Response: Other categories were used for religion and languages, which have been specified in the revision version.

Discussion:
Comment: The section on private sector deliveries highlights issues related to “profit motives and unnecessary procedures,” which can help illuminate existing policy gaps, such as weak regulation, inadequate insurance coverage, and ineffective accreditation systems.
Response: thank you for the feedback, we have included some policy gaps related to delivery in private health facilities.

Comment: While the Aama program is mentioned, it is important to address whether it effectively reduces inequities and to explore why its reach in private facilities is limited.
Response: our study has suggested that Aama program is more equitable than other interventions. However, few private HFs have implemented this program due to various reasons including profit motive from the routine service. Aama program has limited budget ceiling for (unit cost) for the reimbursement for health facilities on implementation of Aama program. If this program is implemented in private HFs, then they cannot charge extra from the service users. If the program is not implemented, they are free to charge to the service users and opt for elective CS even non medically indicated normal deliveries. These arguments have been included in the revised manuscript.

Comment: Additionally, it would be beneficial to include information about systemic gaps in the continuum of care, such as transportation, health workforce capacity, facility preparedness, and the referral system.

Response: thank you for the feedback, we have included these suggestions in the revised manuscript

## Reviewer #3:

Comment: As the current manuscript is prepared using the data of three years, however, Determinants of institutional delivery were assessed through multivariable analysis of the NDHS 2022 dataset. Why the data from remaining two years (2011 and 2016) were not utilized for this outcome?

Response: Our objective was to examine the overall trends of maternal health service utilization over the last three surveys, while understanding the determinants of services utilisation in the recent survey. There are several studies published and examined the determinants of maternal health services utilisation in Nepal using data of NDHS 2011, and 2016.
Comment: (2) After the completion of all procedures we prepare a manuscript, hence, preparing the final manuscript in the past tense is suitable.

Response: corrected as suggested, especially in the methods section.

Comment: (3) "Analyses" or "Analysis", once go through this as per requirement.

Response: Analyses is plural form of analysis, so used as per the need in the manuscript.

Comment: (4) Arrange the key words in the alphabetical orders.

Response: corrected as suggested.

Comment: (5) Line 163: "were categories into", is the tense correct?

Response: thanks for pointing out the editing issues, now it has been corrected.

Comment: (6) "(dis)advantages", this word is little bit uneasy to understand by common viewer.

Response: we revised into marginalized terms which refers the same meaning as socially excluded groups.

Comment: (7) Line 193: "variation inflation factor (VIF)", is it variation or variance?

Response: corrected, it is variance inflation factor (VIF).

Comment: (8) Line 278, Table 1: N = 981, however, total number of male and female is 982, and same is found in other columns as well, do you have any specific reason please?

Response: N was 981. Corrected as suggested.
Comment: (9) Province "Sudurpaschim" has been considered as reference group, if the rationale behind it is described it would be easy for readers.

Response: this was done to make interpretation easy. As the Sudurpaschim is has better results, our focus is to demonstrate the inequity (lack of service utilization), thus we used this reference to demonstrate which other province are left behind in terms of maternal service utilization.

Comment: (10) Regarding age group, both the "<20 years" and "20-24" years are taken as reference groups, was there any specific description?

Response: Same as above, this reference was taken to interpret the findings and make it easy to interpret the results and results as per the outcome variables.
Comment: (11) Digits after decimal are expected to be uniform, however, some findings are described with single digit after decimal.

Response: corrected as suggested.

Comment: (12) Line 29 says you have applied multivariable analysis, Line 189 says you applied multivariate analyses, did you apply both the methods? As they are different methods.

Response: we used multivariable analysis rather than multivariate analysis. Yes, there is difference of both. For instance, multivariable analysis has one outcome variable and many predictor, for instance blood pressure (one outcome) is predicted by age, weight, and smoking status (multiple predictors).But multivariate analysis (more than one outcome variable modelled jointly, and many predictor for instance modeling both systolic and diastolic blood pressure as outcomes, with age and weight as predictors—these two outcomes are analyzed together.

## Reviewer #4:

Comment: This was a secondary data analysis to reflect the maternal health services in Nepal and provides insightful evidence on this sector. Improving maternal health is a national priority in Nepal, supported by the government’s Aama Suraksha program, which provides free delivery care and financial incentives to encourage women to access the maternal health care. Using nationally representative survey data (Nepal Demographic Health Survey of different years), this study examined whether women are receiving the recommended continuum of maternal health services-antenatal care (ANC), delivery in a health facility, and postnatal care (PNC)-and whether there are differences across social and geographic groups.

Response: thank you for the feedback.

Comment: This study found that most women attend their first ANC visit and deliver in health facilities, reflecting progress in service use. However, far fewer women receive timely PNC, and only a small proportion complete all three essential services. Gaps are especially large among women from disadvantaged groups, rural communities, and those speaking Maithili or Bhojpuri. Private facilities are increasingly used for childbirth, including caesarean deliveries, but maternal incentive uptake remains low in these settings. The findings suggest the need for stronger efforts to improve continuity of care, expand incentives, and address barriers faced by disadvantaged women to ensure more equitable maternal health outcomes in Nepal.
The authors have aroused needy health gaps in low resources settings like Nepal, and have presented the Nationally represented data for more generalizability. However, this study needs some revisions to give audience insightful and non-misguided interpretations. The review comments are attached too.

Response: This is what exactly our analysis revealed. A great snapshot of our study findings. Thank you for the compliment.

Review suggestions:

Comment: Title of the slide needs revision, maybe adding years and then rewording might give more clarity. I am not clear on this as “Determinants of institutional delivery” includes the year 2022 NDHS data set only.

Response: corrected as suggested in the title. Our study objective is to identify the trends of major maternal health service utilization while investigating the determinants of those service utilization in the recent survey. This objective was chosen to see the overall trends over the last one decade and to see the crucial factors influencing the service utilization in the recent years. This objective chosen considering the policy point of view and discussed with wider stakeholders (as this paper is based on the further analysis of those NDHS reported lead by ministry of health and Nepal and where most of authors were involved in those further analysis). Other reviewers have also commented on the reasons for the selection of data of NDHS 2022 only to examine the determinants of maternal service utilization. We argue that there are several past studies already investigate the determinants of maternal services using data from NDHS 2011, and 2016. For example, first author and team also identify the determinants of maternal service using data from NDHS 2016. Thus, author team thought better not waste efforts to re-invent the wheel again.

Comment: Line 28 in abstract section: “Completion of all maternal needs” required to be specified as it sounds like it comprises of everything that author has already mentioned (at least four antenatal care (ANC) visits, institutional deliveries, first postnatal care (PNC) within 48 hours of childbirth).

Response: corrected as suggested.

Comment: Line 32-47, Results in abstract section: The paragraph is dense and strings multiple findings together. Breaking into sub-paragraphs by theme might help readers to understand the results summary clearly.

Response: thanks, revised as suggested.

Comment: Findings are described qualitatively (“increased odds”, “reduced rates”) without any quantitative estimates, making it difficult to understand by how much. Missing total subjects included in the analysis, as 2 different aims have different sample size. You have mentioned it in the methods section, but it is not clear if all the samples are included or not in the final analysis.

Response: revised as suggested. It is impossible to include in the abstract given multiple outcome variables included in the analysis. Detailed description is given in the method section of manuscript.

Comment: Line 40: completion rates used here are a bit vague, this may require specific definition in an appropriate space

Response: clarified as suggested.

Comment: Line 43: “women engaged in manual labor” means two different things to me, please reword this phrase to make it more clear on what you are trying to say

Response: corrected for the clarification.

Comment: Instead of increased odds or reduced odds, consider using higher or lower

Response: corrected as suggested.

Comment: Some sentences are very long (esp. lines about multivariable analysis). Breaking them into shorter, sharper statements will improve readability.

Response: corrected as suggested. Complex sentences are split into simple sentences for clarity.

Comment: What are “disadvantaged ethnicities” in line 38?

Response: corrected as suggested. Detailed in the method section.

Comment: Conclusion section (line 48-57), Sentences are long-rephrase for clarity. Recommendations (Lines 54–57) should be specific and findings-driven and reword “nonexistent maternal incentives” to something clearer.

Response: corrected as suggested, thank you.

Keywords (59-60): Please arrange the keywords in alphabetical order as per journal conventions.

Comment: Additionally, consider prioritizing terms based on their frequency and centrality in your study. Ensure consistency with terminology used in the abstract.

Response: corrected as suggested

Comment: Introduction: Line 69-70 says increased but does not explicitly mention what kind of increase (quantitative information is missing)

Response: added some quantitative data to substantiate the qualitative argument.

Comment: Line 73 to 78 and check throughout if missed in remaining sections too: recheck the referenced paper and mention the unit for MMR and NMR numbers

Response: citations were updated, and quantitative data were included.

Comment: Line 74: Citation is out of order, consider reordering. And recheck the reference and the referred article for citation number 21 here, as content does not match with the cited paper

Response: Appropriate citations were added to reflect the content.

Comment: Revise all the citation numbers, also check contents from appropriate references

Response: revised as suggested

Comment: Once abbreviated, it is redundant to abbreviate in the following texts, revise accordingly

Response: revised as suggested.

Comment: Line 83 to 85 mentions the percentages, and it would be nice to learn out of how much (denominator)

Response: revised as suggested. These data were from NDHS 2022. These data were taken from the publication cited in the sentence. It is not common practice to include such detail of others work in the introduction or discussion section. Interested readers can read the cited papers.

Comment: Line 88, not so sure if 10^th^ citation really reflects the information explained in that sentence. Check if referencing and cited sentences are related or not

Response: revised as suggested, reference was checked and edited.

Comment: Line 89: could not understand the meaning of contact coverage? Is there any better way to explain it?

Response: contact coverage refers service users simply visiting t health facilities or health care workers for services, for instance, women completed at least four antenatal care visits (yes or no) but not describe what kind of antenatal care interventions they received in that visit. We have explained on in the sentence. Additionally, authors previous publication is cited here where reader can go and explore on it.

Comment: Line 119, 124: Aama program (maternity incentive program), check the specific name for this program as this is widely popular program too

Response: thanks, we have revised it

Comment: Line 120: Transportation costs meaning? Payment, reimbursement, partial payment? Any specific amount?

Response: it has been revised and included amount for transportation cost provided to the women who received for maternity care visits.

Comment: Authors gave detailed stats (MMR, NMR, inequalities, Aama details, federalism, private sector shifts) before that please state clearly what’s missing in research. Hence, making readers not able to grab the study gap. Revise sequencing the sentences

Response: revised as suggested.

Comment: Equity gaps by wealth, education, ethnicity, and provinces are repeated in different places.

Response: revised as suggested, thank you.

Comment: Ensure terms like *“maternal incentive”* vs. *“maternity incentive program”* are consistent.

Response: its maternity incentive program, revised and made consistent throughout.

Comment: Aims of the study: The aims of the study have been expressed differently in the abstract section and introduction section, as well as need to synchronize with the title. Please revise accordingly

Response: Thank you for the feedback, we have made the study objective consistent throughout.

Comment: Methods: Line: 145-146: cite the link of DHS consistently, this can be listed in the reference list too. If there is specific link to the dataset, use those links

Response: Thanks, revised as suggested.

Comment: Elaborate explanations on study design, settings, study participants (eligibility criteria), sample size including sampling technique. Flowchart is helpful to understand how the participants are included in the study, or detail explanation is required. If different aims have different study settings, explain them clearly too

Response: we used secondary data from NDHSs. This is further analysis of nationally representative cross-sectional household survey data derived that NDHSs 2011, 2016, and 2022. The unit of analysis in this further analysis was women who had a live birth at least one year prior to the survey. detailed methodology of sampling design is included in the original reports. We have briefly touched the sampling methods and data collection in NDHS survey. NDHS collects information related to pregnancy and childbirth from the women who had live birth five years prior to the survey. From that bigger cohort of women, we have included in our sample women who had a live birth one year prior to the survey. We have included this information in the methods section. We have included relevant information in our methods section focusing on what we have done in this analysis rather than what NDHS has done to in their sampling design and data collection.

Comment: Line 149-150: Please justify why only one year prior to each selected NDHS was selected.

Response: this was done to reduce the recall bias. If women had longer windows of recall, then participants might have higher recall bias of the service received. This is one of the strengths of our study given the further analysis of secondary data.

Comment: Mention in separate section or elaborate about the potential confounders.

Response: we have included the available variables in the dataset, selected certain variables that are important for the policy purpose.

Comment: Line 151-152: For 796 (weighted), if weighted meaning weighted, this should be explained in findings than methods section. As weighed analysis is done only after analysis.

Response: it is better to explain what has been done rather than what has found further analysis. we have clarified that our sample size is weighted to avoid the confusion to the readers.

Comment: Study variables: explain dependent and independent variables in separate headings for more clarity. While explaining the variables, refer to the particular aims for better understanding to the readers

Response: thank you, it has been revised as suggested.

Comment: Please explain well how the outcome/dependent variables were measured.

Response: This is the further analysis of NDHS data where outcome variables were based on response of the participants (in our case women) whether they used health services or not (in our case maternal health care visits or CS, or maternity incentive). If they used services, then YES=1, otherwise NO=0.

Comment: Provide rationale for dichotomization of key variables and consider sensitivity checks.

Response: Dichotomisation key variables (ethnicity, wealth status, and education) were based on the theoretical underpinning and comparative privilege in the society and with the argument that marginalized groups (women from poor households) generally have poor access to health services. Similar categorization was done for ethnicity, and education level. These has been described in the methods section. Such dichotomization was useful to create a new variable with multiple forms of marginalization status. This was done based on prior work based on NDHS 2016 data and other similar studies in other similar settings (cited in the method section).

Comment: Line 161 and 162: For the definition of marginalization status, why it has been adapted from the study in India? Mention a line to justify this

Response: First author and team did similar study using data from NDHS 2016, and data from the Ethiopian Demographic and Health Survey 2016. These are cited in our study too.

Comment: Line 162-168, clearly explain which steps are those.

Response: explained and revised.

Comment: The term disadvantage has been used several times, and other terminologies also include this disadvantage terminology, which creates confusion while reading this. This needs to be presented more clearly

Response: we have revised the term throughout and change to marginalization status, which refers to socially excluded groups.

Comment: How is wealth quintile measured? Mention this in detail, since this is also one of the key variables used in the study. You may explain this is S1 table if there is space limitation

Response: This study is based on the data from the NDHSs, where wealth quintiles were measured composite index of several assets at the household level. Our focus here (any further analysis) is what we did rather than what NDHS did to construct the wealth quintile. However, we have little touched how wealth quintiles in DHS survey and cited them. This helps reader to go through the original report how wealth quintiles are measured in the DHS survey.

Comment: Please clearly mention the definition of the four categories of disadvantage status: triple, double, single, and no disadvantages

Response: it was described in the methods section, and fully described in our previous publication, which have been cited here. Brely triple disadvantaged means women who are low wealth status and illiterate and marginalized ethnic groups, double disadvantaged means women with any two of these disadvantaged (any two of poor and illiterate and marginalised ethic groups), single means any of disadvantage of three (any one of poor or illiterate or marginalized ethic group) and do disadvantages means rich and literate and advantaged ethic groups.

Comment: Conceptual framework: While the framework is presented, it is not entirely clear how it was applied to variable selection or analysis. Could not follow how this conceptual framework was applied and coordinated with other sections of the study.

Response: The conceptual framework was a guiding and thinking framework for this paper (see response to another reviewer above). With broader domain of structural factor, intermediary factors, and health system factors, variables were included based on data available in the dataset. Broadly, structural factors are non-modifiable factors (by health system), and health service need to provide those with women of such structural disadvantages. Intermediary factors those can be addressed through modifiable development approach (e.g. reducing travel time etc), and health system factors focus on access and quality of health services (modifiable through health system). We used this framing to guide our analysis and selected variables available in the NDHS dataset.

Comment: Data analysis (line 188-198): The last sentence sounds like author used STATA only for the survey, so make it clear by rewording the sentences

Response: revised as suggested. NDHS survey used other software to analyse the data but, in our analysis, we used Stata.

Comment: The analysis methods are expressed in general structure, please specify which tests were used for which purposes. How many models are used? Which analysis were conducted for aim 1 and which ones for aim 2, explain in detail. Please specify statistical tests specifying the use of it in specific variables.

Response: thanks for this feedback. Proportion test was conducted form the significance test of the trends results while logistic regression analysis was conducted to identify the factors associated with outcome variable. We have clarified these in the revised manuscript.

Comment: For adjusted analysis, please explain which covariates were adjusted

Response: All variables included in the table were included in the adjusted analysis.

Comment: DHS datasets usually have some missingness. Did you exclude missing cases listwise, or apply imputation? This should be mentioned for transparency.

Response: we did not apply imputation as there was no missing data.

Comment: Since the study emphasizes intersectionality (triple/double disadvantage), reviewers may expect clarification on whether interaction terms were tested in regression models, or whether disadvantage status itself captures these interactions.

Response: intersectional variable was created using wealth status, ethnicity, wealth status. The new variable (marginalization status) was corelated variables with the were excluded in the adjusted regression analysis. No interaction terms were tested in the models. Our aims of the analysis were to see the association rather than establishing causal link of independent variables and outcome variables of the study.

Comment: Research ethics: Line 203-204: Does this study need to mention about the informed consent by mothers on behalf of their children? The study participants sentence does not explain it includes children, or it does, explain this clearly in the study participants’ section

Response: we used publicly available de-identified secondary data derived from the NDHSs. Full ethical procedure was explained in the respective report of NDHS analysis. No further ethical approval was needed to conduct this further analysis. For this analysis, we have granted permission to download and use these data (from DHS program webpage). We have included this statement in the manuscript.

Results section:

Comment: Any particular reason to compare national level trend in the findings, if not, discussion section is a better space to do all these comparisons. In the finding section, the study’s findings should be highlighted. Check this for following sections throughout findings section

Response: we investigated the overall trends and trends by intersectional marginalisation status of maternal service utilization among women. These are explained in the finding section. Thanks for the suggestions, we have revised the findings section

Comment: Line 218-219: Requires quantitative information as well.

Response: revised as suggested.

Comment: Line 221-224: what do those statistically significant statements reflect, if authors have used any statistical tests, provide some numbers as well as significant values. Need clarity on what statistical test determined “significance.”

Response: Figures can be more congested if we include much information on them. We have included detailed data including p values in supplementary file. Cut off point for the p value (p<0.05), so if any trends were significant on these cut-off value we have denoted by symbols in different survey years.

Comment: Line 209-227: the findings are more subjective, please explain it in a quantitative way

Response: revised as suggested

Comment: Lin 223: after NDHS looks incomplete. NDHS???

Response: revised as suggested

Comment: Line 242: cs should be capitalized

Response: revised as suggested

Comment: Line 261-268: Could not compare with the presented graph in figure 6.

Response: revised as suggested

Comment: Line 271-276: What is the total sample size for those percentages used?

Response: total sample size is included in the methods section.

Comment: Table 1: Which test gave CI? Mention about this under the table as a “Note”

Response: its chi square test, discussed in the note.

Comment: Re-total % in table one as it required to total to 100%, wherever appropriate

Response: This table represents the column percentage as the distribution of sample. The percentage of all categories of each variable is 100%. Corrected as suggested.

Comment: Line 313 – 333, mention variables those were adjusted. Authors may use the space below table as a footnote. Follow this for the following paragraphs too

Response: all variables included in the table were adjusted in the model.

Comment: Discussion

- Add strength of the study portion
- Reorder the discussion section as follows:
- Main findings (summary in 2–3 paragraphs).
- Comparison with existing literature (situating Nepal in regional/global context).
- Strengths & limitations
- Implications for policy & practice

Response: Revised as suggested.

Comment: In the limitations: the sentences are *dense and technical*. I prefer clear, plain-language explanation of how these limitations might affect results of the study

Response: thank you, revised. The text has been simplified and revised.

Comment: Report other relevant limitations (cross sectional design, self-reported data, no quality-of-care indicator, language and cultural barriers) explaining how it distorts study findings.

Response: thanks, suggestions are included, the suggested limitations have been included.

Comment: Figures: Figure title: Clear label missing

For graphs: Label axes to reduce clutter (avoid repeating % signs).

Figure 2: colors used are less distinct, consider making it more visible

Label the axis for more visual expressions, this will avoid use of several % signs in 1 figure

Figure 4: add title, labels and omit % in the graph.

Response: All figures are **revised** for clarity, including updated colors, labels, and axis adjustments.

## Reviewer #5:

The authors have submitted a technically sound article using the latest data of NDHS. The statistical analysis was done correctly but in some cases few changes can make the results more meaningful. In this manuscript as the figures can’t be added, there was some lack of clarity regarding the conceptual frameworks and few more figures. However, the article is helpful for policymakers to plan for the related activities in future. It can surely be published with minor changes. Limitation and Recommendations on the study titled, " Trends and Determinants of Maternal Health Service Utilization in Nepal: Insights from the Most Recent Three Demographic and Health Surveys".

Comment: **Limitation** Missing Figures and Tables: This makes it hard to verify / understand the results, like trends by marginalization status and conceptual framework. *[Results sections].*

Response: LOS One guidelines suggest including figures in separate files. **Tables** are included in the main manuscript. **Figures** have been placed in a separate file as per the journal's formatting requirements.

Comment: Recommendations: It would show more meaningful results if switched to Relative Risks (RRs) for Common Outcomes: Logistic regression odds ratios are reported, but some outcomes are quite common (>50%), so odds ratios may exaggerate effect sizes. Relative risks could have been more appropriate for those outcomes. *[Methods, Results sections].*

Response: thank you for this feedback. Based on previous studies, we decided to use odds ratios rather than relative risk in our analysis. The odds ratio (OR) is the most widely used effect size measure for categorical outcomes (usually binary) in cross-sectional studies, especially when logistic regression models are used.

Comment: Disadvantaged Categories: The study creates a "disadvantage status" variable by combining education, wealth, and ethnicity into four groups (triple, double, single, or no disadvantages). To do this, it simplifies these factors into two categories each, like splitting wealth into lower 40% (poorest) and upper 60% (richer). This simplification makes the data easier to analyze but loses important details and it treats all poor people the same, even if some are much poorer than others. Because the new variable is made from these three factors, they overlap too much (collinearity), so the study couldn’t include education, wealth, or ethnicity separately in the analysis. This hides how each factor affects things like hospital deliveries on their own, making it harder to pinpoint specific problems or solutions. However, it is included in Limitation section. ***[Methods section].***

Response: thank you for the compliment and for your insights. Yes, due to collinearity of wealth, education, and ethnicity with the new variable — marginalization status — we excluded these three variables in the final model. Since our study's primary objective was to understand service utilization among women with intersectional marginalization status, we have clarified the limitations regarding this exclusion in the Limitation section.

Comment: **Expand the Limitations Section**: Add details on NDHS biases (e.g., "Self-reported data may cause recall bias for ANC/PNC timing") and incentive analysis issues (e.g., "Including facilities where Aama Program was not available may underestimate uptake"). This change can make the study more transparent ***[Limitations section].***

Response: thank you, the limitations have been expanded as requested to provide more transparency about potential biases and the limitations related to the Aama Program analysis.

Comment: **Policy Recommendations**: Recommendations are broad (e.g., Regulate private sector, expand Aama program). The recommendations would benefit if it were sharper, specific and actionable guidance***. [Recommendation Section]***

Response: thank you for the feedback. We have provided **more** specific and actionable policy

## Reviewer #6:

• Comment 1 (Background section):
The statement “Nonetheless, the maternal mortality ratio (MMR) remains high” is vague. Instead of presenting it hypothetically, the authors should provide clear evidence with specific data or figures to support the statements.
Response: corrected as suggested. We have substantiated the argument with quantitative information, including specific data from the latest surveys.
• Comment 2 (Introduction section):
The authors mentioned that “Over the past few decades, Nepal witnessed an increase in household income, wealth status, life expectancy, access to education, and basic health services.” However, to my knowledge, this Annual Report of Nepal, does not measure household income and wealth status. The authors should cite appropriate sources such as the Nepal Living Standards Survey or surveys conducted annually by Nepal Rastra Bank.
Response: The suggested reference has been cited in the revision manuscript. We have now referenced the Nepal Living Standards Survey and Nepal Rastra Bank reports for accuracy.

• Comment 3 (Data analysis section):
The authors reported that they checked the Variance Inflation Factor (VIF) for multicollinearity and excluded some independent variables. It would improve clarity if the authors specified which variables exhibited collinearity and were subsequently removed from the regression models.
Response: The eco-region was removed from the adjusted regression analysis.

• Comment 4 (Ethical section):
The statement “the survey received ethical approval from the ICF Institutional Review Board in the USA and the Nepal Health Research Council, Nepal” is unclear. The authors should write the full form of ICF to avoid confusion, as it could refer to different organizations.
Response: Thanks for the suggested. It has been revised as suggested.

• Comment 5 (Analysis and Results section):
The authors mentioned in statistical analysis section, adjusted odds ratios (AORs) were calculated in bivariate analysis using the Chi-Square test. This is methodologically incorrect because the Chi-Square test does not account for confounders and therefore cannot produce an AOR, whilst only limit on crude (unadjusted) odds ratio (CORs). Logistic regression should be used instead.
Response: thanks for pointing this out. We overlooked this issue. Crude odds ratios were estimated in the bivariable logistic regression analysis. This has been corrected in the revision.

Comment: The authors should also clarify the criteria used for selecting variables into multivariate analysis. In addition, the table headings currently use the generic term “determinants of…”; it would be more precise to state the exact type of analysis performed.

Response: Thanks for the suggestions. Independent variables were selected based on the data availability in the dataset and their significance in the policy context. Additionally, we also referred to previous studies while selecting independent variables. The conceptual framework also guided the selection of variables under each domain of the framework. The revised table headings are now more precise and specify the type of analysis performed.

Reviewer #7:

Comment: The authors have well prepared the manuscript, which is technically sound. The methodological part is well explained according to the NDHS report. Few queries and suggestions regarding variables and typo error have been provided in the attached file below.
The authors have well prepared the manuscript. There are few questions and suggestions from my side.

Response: thank you for feedback

Comment: Line 23: I think the word ‘analyses’ should be replaced with ‘analyses.

Response: analyses is plural forms of analysis. We revised grammar and sentence structure.

Comment: Government has protocol of 24 hours, 3^rd^ day and 7^th^ day.

Response: This is further analysis of secondary data of NDHSs. NDHSs dataset included any PNC visits within 48 hours post childbirth. You are right the government protocol has at least 3 PNC visits within 7 days as mentioned above.

Comment: Results section in abstract: The interpretation of the major findings would be clearer with the numerical value. It is difficult to understand how higher or lower the problem is mentioned. State the percentage or odds ratio where required. Is caesarean section a major variable in the study and a part of maternal continuum?

Response: Quantitative data were included to support the qualitative findings. CS is not part of the continuity of care. In our analysis, three routine maternity care visits were considered for the continuity of care analysis. CS is elective and not all women need it, so it cannot be considered as a continuity of care indicator.

Comment: Line 46: Is there provision of providing maternity incentive by private hospitals?

Response: If private hospitals implement the maternity incentive program, then it is possible. However, there needs to be motivation or a policy framework to encourage the implementation of such programs in private hospitals.

Comment: 236-237: Clarify whether the home delivery is 18 or 19% in 2022.

Response: There was home delivery of 19% in 2022.

Comment: Can you provide reference for the recategorization of independent variables such as ‘marginalized status and disadvantages if any.

Response: The marginalization status was explained in the manuscript, and references have been provided. Studies from India, Nepal (including the first author’s earlier work on 2016 and 2022 data), and the Ethiopian DHS have been cited as references for this categorization.

Comment: 244: Rearrange a sentence as ‘at least a live birth in a health facility’.

Response: revised as suggested.

Comment: 278: Table 1, it would be better to present the distribution of weighted women rather than unweighted or You can present the frequency of both. In the same table, is ‘age in year’ the current age of women or the age of women at childbirth? Please state clearly. If it is the current age, it should be mentioned at first component of the demographic characteristics.

Response: All data presented in the analysis are weighted, unless specifically indicated otherwise. Age refers to the respondent's age at the time of the survey. We have revised Table 1 to reflect this clearly.

Comment: The findings show the doubling of CS in public facilities too, which you haven’t emphasized. Since the size of institutional delivery is too large in public facilities, the percentage might have seen less here. Whereas the delivery of private facilities is less compared to public so that CS seems higher there. Please clearly describe it.

Response: We agree with the reviewer that there is an increase in CS in public facilities as well. The calculation of CS in public and private facilities was standardized with the number of deliveries in those facilities. Our comparison is in percentages rather than the absolute numbers, which adjusts for the size of the population or childbirth in both private and public facilities.

Comment: The interpretation and discussion are okay. Conclusion and recommendations are well written.

Ensure all the components according to STROBE checklist.

Response: Thanks for the suggestion. This paper is based on secondary data derived from a cross-sectional, nationally representative household survey (NDHS). Most of the components of STROBE checklist are followed while conducting and reporting the findings.

## Reviewer #8:

 Comment: The manuscript is well structured and written in standard English. It follows the guideline of PLOS one. The author has used the nationally representative data sets which strengthens the validity of the findings in the study. However, there are some minor grammatical errors which needs to be corrected to improve the flow. Some sentences are too long and needs to be shortened.

Response: Thank you for the comments and suggestions. Grammatical errors have been corrected, and we have shortened long sentences to improve the flow of the manuscript.

## Reviewer #9:

Comment: Inconsistent Sample Sizes
The manuscript's "Methods" and "Results" sections present conflicting information regarding the sample size used for the determinant analysis. The abstract and "Methods" section state that the determinant analysis for institutional delivery was performed on the 2022 NDHS dataset. However, the "Methods" section specifies that the total sample size for the determinant analysis of institutional delivery, delivery by CS, and maternal incentives was 796 women who gave birth at a health facility. In contrast, the "Results" section states that the descriptive analysis of institutional delivery was based on 981 study participants. This discrepancy in sample sizes between the methodology and the results section for the same analysis is unclear.

Response: Total sample size for the women included in the analysis was 981. As this study has four outcome variables: Institutional delivery (sample was all women who had live birth one year prior to the survey i.e. 981), institutional delivery in private health facilities (among all women who had institutional delivery i.e. 796), women who had CS (among all women who had institutional delivery i.e. 796), and women who received maternity incentive (among all women who had institutional delivery i.e. 796). Descriptive analysis of all outcome variables is included in the supplementary file (S2-S5).

Comment: Unclear Explanation of the "Disadvantage Status" Variable
The manuscript explains that a new variable, "disadvantage status," was created by combining three background variables: education, wealth status, and ethnicity. It details the process of dichotomizing these variables and then merging the categories. The final variable is said to have four categories: triple, double, single, and no disadvantages. However, the description of how the categories with "at least one form of disadvantage" and "two forms of disadvantage" were merged is confusing and does not clearly explain which specific combinations of the original eight categories were merged to create the final four. This lack of clarity makes it difficult to replicate the analysis.

Response: When creating a new variable by combining three variables with two categories each, there can be 8 possible categories (see paper by the first author <https://doi.org/10.1186/s12889-021-11142-8>). Due to small cell sizes in some categories, we merged categories with at least one form of marginalization into one group, and similarly, at least two forms of marginalization into another group. Therefore, we ended up with four categories in our analysis.

## Reviewer #10:

Comment: Overall Impression of the Paper: The paper looks well-structured and has provided a glimpse of health inequity and its effects in one of the most important areas, maternal service utilization.

Response: thank you so much.

Comment: Strengths of the Paper: Wise use of NDHS data that represent the whole nation has provided a good rationale and aim

Response: thank you so much.

Areas to improve:

Comment: Abstract:The abstract looks quite lengthy, consider it concise.

Comment: Methods: In the Methods section (line number 151-153) under data sources, the sample size and weighting could be explained quite a little more to make the readers unfamiliar with this concept clearer.
Response Thank you; it has been clarified in the Methods section. As the DHS uses a multi-stage clustered complex sampling design, we used the “svy” command in Stata, which accounts for the survey design, sampling weights, and adjusts for clustering effects in the sampling design.

Comment: In the study variables under methods, section, explanation of outcomes and exposures could be done in separate paragraphs under these headings to make the picture clear.
Response: thank you, revised as suggested

Comment: Provide reference for advantaged ethnicities.
Response: revised as suggested and provided the reference

Comment: Creation of marginalization status and disadvantaged status could be better explained using a flowchart. Figures are yet to be inserted, so it makes the concept cloudy at places.

Response: We have cited our earlier publication on how intersectional variables can be created. We used a similar approach in this paper as well.

Comment: Results: The results are well presented but contain many tables, consider merging the tables where relevant. Also highlight the major findings in tables so that the navigation gets easier. If possible, show 2-3 results in a single graph using multiple colours to indicate different findings’ trend and provide indices.

Response: Thank you for these practical suggestions. We have merged relevant figures and tables when appropriate or merging of data make sense.
