# Supplementary material for: Unpacking equity trends and gaps in Nepal’s progress on maternal health service utilization: Insights from the most recent Demographic and Health Surveys (2011, 2016 and 2022)
Source: PLoS One. 2025 Nov 20;20(11):e0337434. doi: 10.1371/journal.pone.0337434 (PMC12633875; doi:10.1371/journal.pone.0337434)
Supplement: Attachment — Submitted filename: review_maternal 2nd.docx [file pone.0337434.s009.docx]

The authors have well prepared the manuscript and incorporated few suggestions given earlier. There are still few questions which are unanswered.

Abstract: How can you conclude this statement in Line 54-56 (……..worsens financial burden)? I don’t see the same arguments in Conclusion of the study. Please make sure it is not overstated.

Please explain how did you assess the PNC visit within 48 hours. Government has protocol of 24 hours, 3^rd^ day and 7^th^ day. Did you combine 1^st^ and 2^nd^ PNC visits?

Since, there is no provision of maternal incentives providing by all private facilities, is it fair to make a comparison on rise or decline of up taking incentives in private and public facilities?

There has been a revision on providing maternal incentives such as Rs 1000 in Terai, 2000 in Hills and 3000 in mountain. So, it would be nice to state the updated provision too in introduction part Line 146-150.

Some of the results are found repeatedly stated in discussion section. Avoid redundancy. Repetition of same thing may not be interesting to readers. Try to present your findings in a simpler way.

The authors have analyzed determinants of maternal service utilization individually in multiple levels; however, I couldn’t find if the determinants of continuum of maternal care were analyzed. Since the complete uptake was found only 59% so it would be great to know what were the factors associated with it.

In line 621-623, We limited our sample for those women who had at least one live birth one year **prior to the survey to reduce the survey**. Is it correct?

The conclusion and recommendations are well written. Please check again for typo error.
